# Cohort profile for the MASTERMIND study: using the Clinical Practice Research Datalink (CPRD) to investigate stratification of response to treatment in patients with type 2 diabetes

**DOI:** 10.1136/bmjopen-2017-017989

**Published:** 2017-10-12

**Authors:** Lauren R Rodgers, Michael N Weedon, William E Henley, Andrew T Hattersley, Beverley M Shields

**Affiliations:** 1 Institute of Health Research, University of Exeter Medical School, Exeter, UK; 2 Institute of Biomedical and Clinical Science, University of Exeter Medical School, Exeter, UK; 3 Department of Diabetes and Endocrinology, Royal Devon and Exeter NHS Foundation Trust, Exeter, UK; 4 NIHR Exeter Clinical Research Facility, University of Exeter Medical School, Exeter, UK

**Keywords:** general diabetes, primary health care, cohort studies, electronic medical records, hypoglycaemic agents

## Abstract

**Purpose:**

This is a retrospective cohort study using observational data from anonymised primary care records. We identify and extract all patients with type 2 diabetes and associated clinical data from the Clinical Practice Research Datalink (CPRD) to inform models of disease progression and stratification of treatment.

**Participants:**

Data were extracted from CPRD on 8 August 2016. The initial data set contained all patients (n=313 485) in the database who had received a type 2 diabetes medication. Criteria were applied to identify and exclude those with type 1 diabetes, polycystic ovarian syndrome or other forms of diabetes (n=40 204), and for data quality control (n=12). We identified 251 338 patients for inclusion in future analyses of diabetes progression and treatment response.

**Findings to date:**

For 6-month response to treatment, measured by change in glycated haemoglobin (HbA1c), we have 91 765 patients with 119 785 treatment response episodes. The greatest impact on reduction of HbA1c occurs with first-line and second-line treatments, metformin and sulfonylurea. Patients moving to third-line treatments tend to have greater weights and higher body mass index. We have investigated the impact of non-adherence to commonly used glucose-lowering medications on HbA1c. For baseline-adjusted HbA1c change over 1 year, non-adherent patients had lower HbA1c reductions than adherent patients, with mean and 95% CI of −4.4 (−4.7 to −4.0) mmol/mol (−0.40 (−0.43 to −0.37) %).

**Future plans:**

Findings from studies using these data will help inform future treatment plans and guidelines. Additional data are added with updates from CPRD. This will increase the numbers of patients on newer medications and add more data on those already receiving treatment. There are several ongoing studies investigating different hypotheses regarding differential response to treatment and progression of diabetes. For side effects, links to Hospital Episode Statistics data, where severe events such as hypoglycaemia will be recorded, will also be explored.

**Strengths and limitations of this study**

- Different treatments for type 2 diabetes have different mechanisms of action. At present it is unknown which patients will respond best to a particular therapy.
- The Clinical Practice Research Datalink (CPRD) is the largest validated primary care longitudinal health record database in the world and a potentially valuable resource to investigate individual response to treatment in patients with type 2 diabetes.
- This protocol details a method for extracting and cleaning data relating to therapy and biomarkers for studies of type 2 diabetes using CPRD.
- Data from the study can inform models of disease progression and response to therapy.
- There are small patient numbers on newer treatments, such as sodium-glucose co-transporter-2 (SGLT2) inhibitors; however, as the study is ongoing, these will increase.

## Introduction

Understanding the clinical factors that are associated with response to glucose-lowering therapy in type 2 diabetes could form the basis of selecting subgroups of patients who are most suited to a specific treatment, an approach known as ‘stratified’ or ‘precision’ medicine. Type 2 diabetes is an area of medicine where a stratified approach to treatment may be of particular interest because there are differences in the underlying pathophysiology between patients, marked variations in drug response and diabetes progression, and a number of different glucose-lowering therapies with different mechanisms of action. Newly diagnosed patients with type 2 diabetes are usually prescribed metformin as a first therapy. There are at least six other classes of treatment available, for second-line and third-line therapies. Present treatment guidelines do not give information on which subjects will respond best to specific therapies, so decisions about which drug to prescribe are usually based on cost, possible side effects and physician preference.^1^ If we understood the patient characteristics that are predictors of response, then treatment selection could be better informed.^2 3^ The huge volumes of data found in electronic health databases may provide a powerful resource for the identification of stratified treatment approaches in type 2 diabetes.

Using electronic databases is key to collecting evidence to inform potential stratification in type 2 diabetes. The Clinical Practice Research Datalink (CPRD) is the largest validated primary care longitudinal health record database in the world,^4^ making it ideal for analysis of clinical factors associated with response to type 2 diabetes medications. CPRD currently includes records from general practices across the UK, with over 17.1 million patients with usable data.^5^ The database contains information on patients’ demographics (age, sex and so on), medical symptoms and diagnoses, biochemistry results, prescribing information, and lifestyle factors such as smoking and alcohol consumption. Using these data we can assess if different glucose-lowering drugs have different factors that predict response. The longitudinal nature of the CPRD data will also allow models of disease progression and time to failure of therapy to be developed.

There is currently no established framework for reporting protocols for observational studies with electronic health data. There are standard and clear guidelines for reporting clinical trials and systematic reviews including preregistration. CPRD requires considerable coding to produce an analysis-ready data set, and there are many opportunities for differences in interpretation of definitions and different ways of extracting core data. Researchers have reported the need for greater transparency in planning, data quality and reporting studies of observational data.^6–9^ Schuemie *et al*^10^ report that results from observational studies cannot be replicated and cites two studies using the same database over the same period that found different results. The Strengthening the Reporting of Observational Studies in Epidemiology (STROBE) guidelines are set for observational studies, which an electronic health data study would be classed as; however, these guidelines are not entirely applicable to this type of study.^11^ Recently the STROBE guidelines have been extended specifically for studies using routinely collected healthcare data in the REporting of studies Conducted using Observational Routinely collected Data (RECORD) statement.^12^ We therefore have produced this cohort profile to ensure transparency of our approach, marking against the STROBE/RECORD recommendations where applicable, to enable replication of any results (checklist available in online supplementary file 1).

10.1136/bmjopen-2017-017989.supp1 Supplementary file 1

## Cohort description

### Data set

We requested CPRD data from all patients who had been prescribed one of the following diabetes medications: metformin, sulfonylurea, dipeptidyl peptidase-4 (DPP4) inhibitor, glucagon-like peptide 1 receptor (GLP1-R) agonist, thiazolidinedione or sodium-glucose co-transporter-2 (SGLT2) inhibitor, with drugs identified from the products file using British National Formulary (BNF) codes for non-insulin diabetes therapies and identifying further matches by searching both generic and trade names for these drugs. We excluded medications that were not recommended by the National Institute for Heath and Care Excellence from our final data set (ie, guar gum). Data were extracted on 8 August 2016 and contained records from 313 485 patients from up-to-standard practices. Three authors (LRR, BMS, MNW) extracted the data using different software (R V.3.0.2, Stata V.13 and Unix shell scripts and SQL). The final data sets and extracted values were compared to minimise errors in the data extraction process. If there were differences, the extraction coding was examined for errors or omissions.

### Inclusion/exclusion criteria

**Figure 1:**
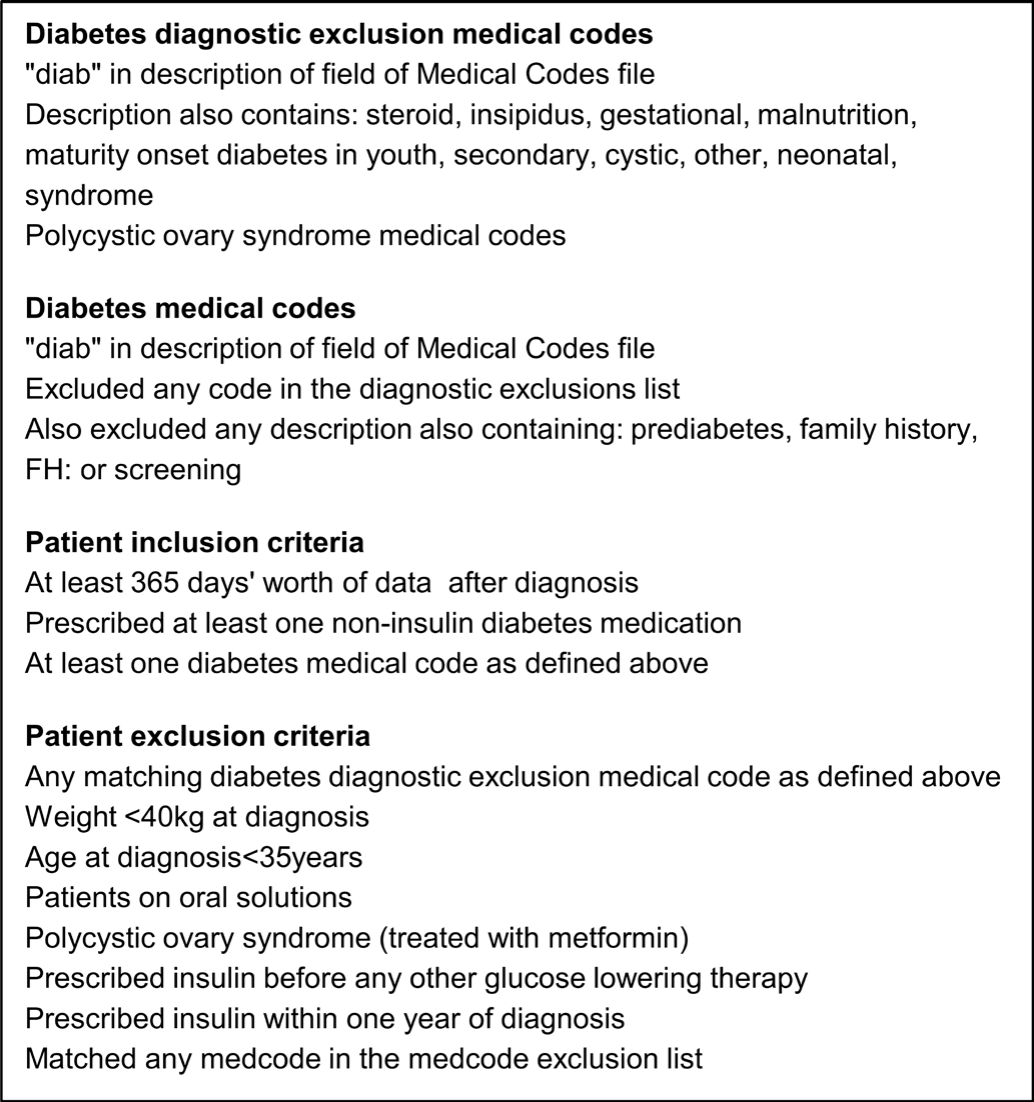
Study population inclusion and exclusion criteria. FH, family history.

**Figure 2:**
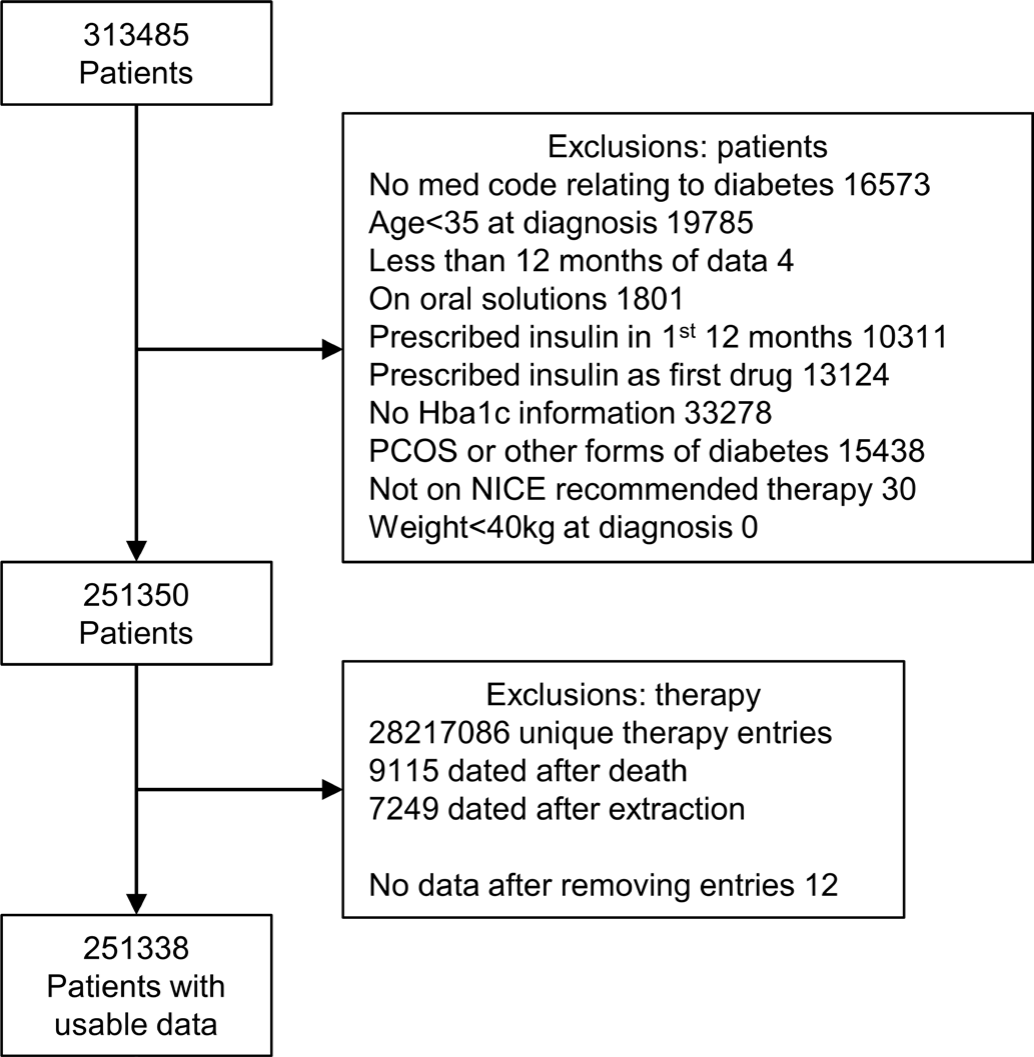
Flow of patients through initial data extraction. HbA1c, glycated haemoglobin; NICE, National Institute for Heath and Care Excellence; PCOS, polycystic ovary syndrome.

The purpose of the data extraction was to identify all patients with type 2 diabetes; we therefore wanted to remove other forms of diabetes that may still be in the data set. The study inclusion and exclusion criteria are listed in figure 1, and the flow of patients through the extraction process in figure 2. The data set obtained after this extraction process will consist of patients with type 2 diabetes; information on initial and repeated treatment regimens will be available. Summary data are provided in (online supplementary file 2). Further refinement will be required to extract a usable data set appropriate to a study, for example change in glycated haemoglobin (HbA1c) at 6 months where we will require patients to have a period of stable therapy prior to starting new medication and have taken new medication for a sufficient period of time to have an effect. Patients with type 1 diabetes were removed from the data by excluding patients with an age at diagnosis <35 years, or patients only treated with insulin, patients whose first therapy was insulin or patients who were prescribed insulin within 1 year of diagnosis. Read codes for type 1 diabetes were not used as there are known problems with coding errors.^13^ Other forms of diabetes, such as steroid-induced, gestational or maturity onset diabetes of the young, were excluded using specific medical codes. Medical codes for family history or scoring test results (entity types 87 and 372, respectively) and with the keywords family, FH, screening and prehistory were excluded from the inclusion medical codes list. Any patients who did not have a diabetes medical code in their records were excluded. Patients on oral solutions were also excluded as adherence and dose calculations for these patients were likely to be different from other patients, introducing potential confounding as to why those patients were on oral solutions and could have different responses/adherence rates.

10.1136/bmjopen-2017-017989.supp2 Supplementary file 2

### Patient characteristics

Age at diabetes diagnosis and duration of disease are key criteria associated with severity of disease and therefore likely to affect response. Date of diabetes diagnosis is not clearly defined in CPRD, so we devised a definition based on medical codes, prescriptions and HbA1c results.

The date of diabetes diagnosis was defined as the earlier of date of first prescription for a non-insulin diabetes therapy, first HbA1c result >47.5 mmol/mol (6.5%) or first diabetes diagnostic medical code. Diabetes diagnostic codes used were as defined in the inclusion medical codes list, with entries restricted to diagnosis entries only (consultation type 3). If the diagnosis date is within 91 days of the current registration date, we assume that the patient had been diagnosed prior to registration with the practice and diagnosis date is therefore unknown. To reduce historic date entry errors, we remove the medical code date if it matches patient year of birth or is of the format 01–01-XXX and the date is pre-first registration date. If the medical code date is before the first registration date and the HbA1c and therapy date is over 2 years after the current registration date, we ignore the medical code date. Ages at diagnosis and at start of treatment are calculated as appropriate given diagnosis, treatment start dates and year of birth.

## Biomarker, covariate and outcome data

### Data cleaning

To ensure that we had quality control in our data, we have set criteria for data cleaning. In the therapy files of diabetes prescriptions, any records after the date of death and date of data extraction were removed. The time period of a patient in the data set was determined by transfer in/current registration date, transfer out, date of death and date of data extraction. Where possible we identified points of data entry errors, such as biomarker values outside sensible clinical limits or obvious errors in drug quantities. In our data there were 5 259 645 weight and 5 188 426 body mass index (BMI) values. Using the median of recorded patient height after the age of 18,^14^ for any date which had a weight but no BMI recorded we calculate BMI (n=43 890), and similarly for BMI but no weight value (n=1935). Any instances of data cleaning are detailed below; no other imputations were conducted.

### Blood test results

Blood test results were extracted for use in both determining patient response to treatment (using HbA1c) and as potential predictors of response (lipids, high-density lipoprotein cholesterol, low-density lipoprotein cholesterol, triglycerides) and renal function (creatinine). Table 1 lists quality control measures that were applied to biomarker data. Limits were defined after consultation with clinicians as clinically plausible, including allowing for particularly high or low values, for each biomarker. Entries outside these limits were considered data entry errors and therefore excluded. Conversions between different units were carried out using standard formulae to ensure all measures were in the same units. Where multiple entries on the same day for the same biomarker were found, we took the average.

**Table 1:** Biomarker limits

Biomarker	Units	Limits	Entity	Units code (data3)
HbA1c	mmol/mol, %	(20, 195) mmol/mol (3.9, 20) %	Excluding 213	0, 1 (%), 61 (%), 97 (mmol/mol)
Weight	Kg	(40, 350)	13	
BMI	Kg/m^2^	(15, 100)	13	
HDL cholesterol	mmol	(0.2, 10)	175	0, 96
LDL cholesterol	mmol	(0.1, 20)	177	0, 96, 110 142
Triglyceride	mmol	(0.1, 40)	202	0, 96
Creatinine	μmol	(20, 2500)	165	0, 96, 142, 138, 99
ALT		(0, 200)	155	0, 61, 127, 164, 277
Glucose	mmol/L	(2.5, 30)	213, 274	0, 61, 96

Entity and data3 are fields in the CPRD data. Unit code 0 refers to missing units.

ALT, alanine aminotransferase; BMI, body mass index; CPRD, Clinical Practice Research Datalink; HbA1c, glycated haemoglobin; HDL, high-density lipoprotein; LDL, low-density lipoprotein.

The main outcome of interest in our study was change in HbA1c from baseline. A keyword search was done on the list of medical codes to identify when tests of HbA1c levels were conducted. Two researchers (BMS and SH) carried this out independently and compared results. Data were extracted from the test results file matching the HbA1c medcodes. Entries with entity 213, identifying glucose, were excluded. Our study will report HbA1c in mmol/mol, and all entries that are recorded in percentage units are converted to mmol/mol using standard formula. HbA1c entries prior to 1 January 1990 were removed from the data set; HbA1c was not used clinically before this, and the dates are more likely to be data entry errors than genuine dates.

We extracted all entries with units mmol/mol, per cent and missing units. Entries with other units were deemed errors. For all missing units we assumed that values ≥3.9 and ≤20 were percentage and values >20 and ≤195 were mmol/mol. We also assumed that percentage values above 20 were misreported mmol/mol and mmol/mol values <20 were misreported percentages and adjusted the values accordingly.

### Drug start and stop criteria

To investigate the effects of new therapies, we defined periods of stable treatment regimens by identifying breaks in medication both of new and existing therapies. Defining start, stop and treatment breakpoints was crucial for consistency for time periods on treatment across the different drugs. For some patients there could be a long break between prescriptions of a given drug, and therefore clinically this would be considered stopping and restarting the drug.

Gaps of >6 months (183 days) were used to indicate stopping a drug. Start dates for a drug were defined if there was no other prescription for that drug in the previous 6 months. Patients must have at least two prescriptions for the drug in the 6 months from the first prescription. A patient was considered to have stopped taking a drug if there was no new prescription of that drug in the 6 months following the last prescription.

### Initial response 6 months and 12 months after commencing therapy

An important outcome is initial response to therapy measured by change in HbA1c. We extracted the first instance of a new therapy with start and stop dates as defined earlier. To ensure that the response was due to a new therapy, we defined a period of stable therapy as no changes in treatment regimen in the 3 months (91 days) prior to the first prescription of the new therapy (starting or stopping any other diabetes medication), and the end of the period as the earliest of either the last prescription date or the last prescription date before another diabetes medication is started or stopped (a break in treatment regimen). Most patients will have a therapy added to their existing treatment regimen and will be on polytherapy; this is standard treatment protocol for patients with type 2 diabetes. The addition or change in therapy is because the existing medication regimen has failed. Defining a stable period of treatment prior to adding a new therapy will ensure that response is attributed to the therapy of interest and not confounded by influence due to a reaction, addition or subtraction of other diabetes medications.

Patients on insulin as one of their other diabetes medications were excluded from analyses. Patients on DPP4 inhibitors and thiazolidinedione as monotherapy were excluded as these were considered unusual treatment regimens. We excluded any therapies starting within 3 months of the current registration date. This ensured that we were not missing any prescriptions that may have occurred before registration. We cannot be sure that registration is the start of their treatment; we may be missing prescriptions due to a break in registration with the practice or a new patient at the practice who has previously been on the treatment. Patients who had a duration of diabetes of less than 1 year are excluded from analyses; their behaviour during this first year is expected to be different from those with established diabetes. Those on thiazolidinedione or gliptin as monotherapy were also excluded.

Baseline HbA1c was taken as the closest value to the first prescription date of a particular therapy between 6 months prior and +7 days of that date. Longer time periods showed changes in the biomarker values. Six-month and 12-month responses were the closest HbA1c±3 months (91 days) of either 6 months or 12 months after the first prescription date for that drug. A 3-month window was chosen as we would see an effect on HbA1c after 3 months, and the effect at 6 and 12 months would still be seen in the 3 months after the 6-month and 12-month points. If there was a change in treatment regimen (starting or stopping another diabetes medication) between 6 and 12 months, we did not extract HbA1c values past the date of treatment regimen change. This was to ensure that any change in HbA1c was due to the therapy of interest and not to other changes in glucose-lowering therapies.

Our ±3 months’ allowance for HbA1c response means that potentially we would have an HbA1c response from patients after 3 months and 9 months for 6-month and 12-month responses, respectively. We therefore also included time on therapy for 6-month response as at least 3 months on therapy and for 12-month response at least 9 months (274 days) on therapy. Where the patient was on therapy for less than 6 or 12 months, their response HbA1c values were taken between 3 months from the start of therapy and 3 months after their last prescription or up to a change in treatment regimen, whichever is the earlier.

Baseline, 6-month and 12-month measures for other biomarkers as listed in table 1 were extracted using the same process as for HbA1c.

### Adherence (medical possession ratio) and dose

The medication possession ratio (MPR) is used to assess adherence to medication.^15^ MPR is reported as a percentage and defined as the amount of medication the patient has available to them given the amount of time it is prescribed for. MPR is based on issued prescriptions only; no encashment data are available. Dose is a weighted average of prescribed doses over the time period of interest. To obtain MPR and dose, we first calculate the number of days of tablets for each prescription. MPR and dose are not calculated for GLP1 or insulin drugs as they are dispensed in pens and injections, and therefore daily dose is more difficult to determine.

Some data cleaning is required prior to calculating MPR or dose. The number of days of available tablets can be calculated using either the number of days field, or by dividing the daily dose by total quantity prescribed. Missing values can be calculated using any two of the three values. Daily dose is defined as the number of tablets taken each day. Daily dose is sometimes recorded as milligram rather than dose. Where dosage is recorded as 40, 80, 160 or 240 and the product name contains 80 mg, we convert to a number of tablets per day (daily dose). Similarly for daily doses of 500, 1000, 1500 and 2000 where the product name contains 500 mg, the daily dose is calculated. Total quantities of treatment prescribed over 365 (0.1% of 33 671 394 prescriptions) were assumed to be data entry errors and changed to 0 (missing). Quantities of 556 for metformin prescriptions are also considered data entry errors and corrected to 56. For multiple prescriptions of the same drug class on the same day, we took the average of the number of days, quantity and dosage.

MPR and dose are calculated only where we had an uninterrupted stable treatment period of at least 12 months. The time period for MPR was drug start (first prescription) to the date of the first prescription after 365 days on the drug. The adherence measurement was the number of days of available tablets divided by the number of days between the start of treatment and the first prescription after 365 days, expressed as a percentage.

The daily dose was recorded as 0 in 30% of the 33 671 394 diabetes prescription records. These were often initial prescriptions where patients’ dosage instructions may be titrated. The number of days of tablets cannot be calculated without a daily dose. Where this occurred, we removed the prescription from the adherence calculation, and the time between the start of the prescription with zero daily dose and the next was removed from the denominator. At least three prescriptions with non-zero daily doses were required to calculate the adherence. This was deemed to be sufficient information to perform a calculation of dose. Patients were considered adherent to their medication if the adherence value is between 80% and 120% (inclusive). Examination of the data shows that adherence above 120% is likely caused by unusual prescription regimens and incorrect quantities entered.

Similar to the MPR data, the time period for dose calculations was drug start date to the last prescription before the 12-month HbA1c response value. For each prescription the percentage of maximum dose was calculated by multiplying dosage (drug strength) by the daily dose and then dividing by the maximum possible dosage. The maximum dose was taken from the BNF. We extracted the maximum dose and a weighted mean dose (weighted by days of tablets issued) over the time period. As with adherence we required at least three non-zero daily dose values to calculate dose information.

## Findings to date

**Figure 3:**
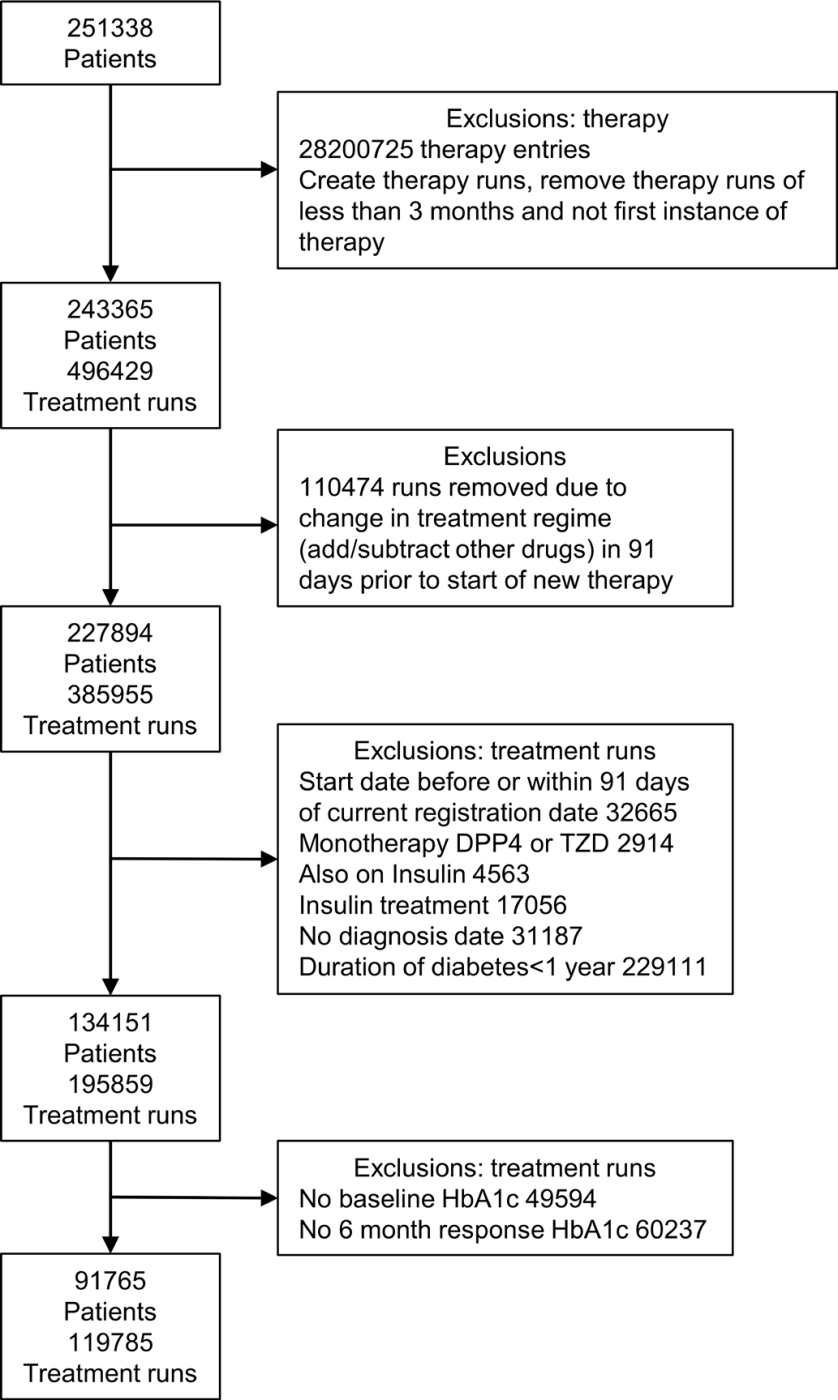
Extraction of patients for data set of 6-month response to initial treatment with new therapy as measured by change in glycated haemoglobin (HbA1c). DPP4, dipeptidyl peptidase-4;TZD, thiozolidinedione.

In our model of initial response to medication as measured by change in HbA1c, 91 765 patients had information on 119 785 first instances of therapy (figure 3). Patients can contribute to more than one therapy response but not at the same time. Table 2 contains baseline information for each medication and the change in HbA1c at 6 months who had baseline and 6-month HbA1c recorded. Patients were required to have been on stable therapy prior to start of therapy, and all prescriptions relating to the treatment run at least 91 days after the current General Practitioner surgery registration date. The majority of treatment information is on first-line and second-line medications (metformin and sulfonylurea), and we find the greatest reduction in HbA1c at 6 months on these medications. Patients moving on to third-line therapies (thiazolidinedione, DPP4 inhibitor and so on) do so later in their disease, shown by longer duration of time since diagnosis. These patients tend to be heavier with greater weight and BMI.

**Table 2:** Baseline information and change in HbA1c at 6-month response

Drug	N	Gender (male) (%)	Age at diagnosis (years)	Duration of diabetes (years)	Percentage on other drugs	Calendar year of start	Baseline HbA1c (mmol/mol (%))	6-Month change in HbA1c (mmol/mol (%))	Baseline weight (kg)	Baseline BMI
Metformin	46 802	58.90	61.1 (10.7)	4.5 (3.5)	26.40	2007.0 (5.0)	64.5 (16.9) (8.0 (1.5))	−6.6 (13.8) (−0.6 (1.3))	88.5 (18.9), 37 897	31.2 (6.1), 37 832
Sulfonylurea	32 751	59.10	59.4 (11)	5.1 (3.5)	72.00	2006.3 (4.7)	64.5 (17.7) (8.0 (1.6))	−6.6 (14.5) (−0.6 (1.3))	89.7 (19.9), 25 599	31.4 (6.2), 25 545
Thiazolidinedione	17 953	59.80	57.1 (10.1)	6.5 (4.3)	100	2006.0 (3.0)	68 (15.8) (8.4 (1.4))	−6.4 (11.9) (−0.6 (1.1))	90.6 (19.5), 14 341	31.7 (6.1), 14 333
DPP4 inhibitor	15 620	60.50	56.5 (10)	7.9 (4.9)	100	2011.8 (2.0)	67.6 (15.9) (8.3 (1.5))	−3.7 (11.5) (−0.3 (1.1))	93.7 (20.3), 12 440	32.7 (6.3), 12 425
GLP1-R agonist	2906	56.50	51 (8.5)	7.8 (4.3)	97.80	2011.2 (2.1)	69.4 (17.7) (8.5 (1.6))	−3.6 (12.7) (−0.3 (1.2))	109.9 (22), 2622	38.1 (6.9), 2621
SGLT2 inhibitor	1620	62.70	51.8 (8.2)	8.5 (4.8)	97.80	2014.6 (0.8)	70.9 (16.5) (8.6 (1.5))	−5.3 (11.3) (−0.5 (1.0))	99.7 (20.2), 1360	34.4 (6.5), 1359
Acarbose	1338	55.80	58.2 (10.4)	7.1 (4.3)	92.20	2000.8 (4.3)	72.4 (18.2) (8.8 (1.7))	−4.1 (13.2) (−0.4 (1.2))	86.2 (19.5), 979	30.5 (6.1), 976
Glinide	795	56.20	56.4 (10.3)	6.2 (4.2)	83.50	2004.5 (3.9)	67.9 (16.9) (8.4 (1.5))	−2.8 (12.1) (−0.3 (1.1))	90.1 (19.8), 626	31.7 (6.3), 625

N is the number of patients starting the therapy who have baseline and 6-month HbA1c recorded. Mean (SD) or mean (SD) N (where N is smaller than column 2) reported unless otherwise stated.

BMI, body mass index; DPP4, dipeptidyl peptidase-4 inhibitors; GLP1-R, ggucagon-like peptide 1 receptor; SGLT2, sodium-glucose co-transporter-2.

The impact of taking glucose-lowering medications intermittently, rather than as prescribed, on HbA1c has not been previously studied. We found that low adherence to commonly used glucose-lowering medications, as measured by the MPR, was associated with smaller reductions in HbA1c.^16^ Data in this study were extracted in August 2014. The same protocol was used to define patients and outcomes as at 6-month response. This cohort of patients was required to have response data at 12 months, as we defined adherence based on at least 12 months of data. There were 32 634 patients with 38 100 treatment instances of duration at least 1 year taken from CPRD data with Hba1c measured at baseline and 12 months. Approximately 13% of CPRD patients were non-adherent to medication, varying across different medications. For baseline-adjusted HbA1c change over 1 year, non-adherent patients had consistently lower HbA1c reductions than adherent patients, with mean and 95% CIs of −4.4 (−4.7 to −4.0) mmol/mol (−0.40 (−0.43 to −0.37) %). The results from the CPRD data were replicated in the GoDARTS (Diabetes Audit and Research Tayside) database, a Scottish database of 9400 patients with diabetes containing primary care, pharmacy and hospital data.

## Strengths and limitations

The CPRD is a superb resource reflecting everyday clinical practice and is an ideal data set to identify factors associated with drug response in a common condition like type 2 diabetes. However, considerable data cleaning is required and rules developed to extract non-entered data, like date of diabetes diagnosis. The work on response to glucose-lowering therapy mainly requires prescription information and biochemistry such as HbA1c; both of these are directly downloaded, greatly reducing errors and increasing coverage. However the potential for errors still exists especially for fields that are entered by hand and different individuals may use different codes for the same clinical event. This means cleaning and manipulation of the data are required, and this paper aims to outline our rationale and methods.

The main purpose defining our cohort data selection is to ensure transparency and clarity in extracting response data on patients with type 2 diabetes in CPRD. We have laid out clear guidelines for extracting and refining data, as illustrated in figures 1–2. To ensure within-study quality of data extraction, we have minimised errors due to coding by using multiple researchers to extract data in parallel. This also proved to be a useful exercise in different researchers finding additional criteria to add to our inclusions and exclusion lists. Our medical and therapy codes are added to the (online supplementary appendix) for other researchers to use.

Electronic healthcare databases lack the randomisation to therapy of a randomised control trial, but real-world effectiveness and utilisation of therapies can be assessed.^4^ Benefits from the large numbers in the databases include the ability to study less common events and the immediate availability of long-term follow-up data. Response to therapies can be studied where it is not possible to conduct a clinical trial due to cost, ethical considerations or impracticalities. However, as this is ‘real life’ data, it may be subject to bias and confounding. For example patients are not randomly allocated a treatment as in a clinical trial. Population heterogeneity, non-linearity of predictors and missing data are also a potential problem. For example, a linkage with Hospital Episode Statistics (HES) data can provide information on some side effects such as hypoglycaemia and cardiovascular disease, but this is limited to England only. For each study using the data, these issues will need to be explored and sensitivity analyses conducted where appropriate to explore how robust the models are and which models need exclusions or adjustments. Methods to deal with unmeasured confounding in non-randomised longitudinal studies will be explored,^17^ such as the prior event rate ratio,^18^ which uses outcomes prior to treatment. Although the data are subject to bias, the results from an analysis of CPRD data will give a pragmatic analysis of practitioner choices and produce generalisable results; any approach to stratification will be applied in ‘real world’ care.

Results from the studies using CPRD data will be compared with other observational data (GoDARTS database) and trial data, now available through online portals such as Clinical Data Study Request and Yale University Open Data Access YODA. Where possible, models developed in one population will be fitted to data from other populations. The direct and indirect comparisons across different data sets will help to validate any findings.

We have detailed our method for defining and extracting initial response to diabetes therapies from patients with type 2 diabetes to inform stratification. This provides a clear protocol for other researchers to reproduce, evaluate and extend work on type 2 diabetics in electronic healthcare databases.
