# Supplementary material for: Cohort profile for the MASTERMIND study: using the Clinical Practice Research Datalink (CPRD) to investigate stratification of response to treatment in patients with type 2 diabetes
Source: BMJ Open. 2017 Oct 12;7(10):e017989. doi: 10.1136/bmjopen-2017-017989 (PMC5652624; doi:10.1136/bmjopen-2017-017989)
Supplement: Supplementary file 2 [file bmjopen-2017-017989supp002.pdf]

SUPPLEMENTARY MATERIALS

Supplementary Table 1

Drug	N prescriptions	N patients with at least one prescription	Gender (Male)	Duration of diabetes at first prescription (yrs)*	Age at first prescription (yrs)	Time between first and last prescription (yrs)	Calendar year of start	Baseline HbA1c (mmol/mol [%])	Baseline weight (kg)	Baseline BMI
Metformin	13521225	232885	56.9%	2.6(3.6)<br>212426	62.5(12.0)	6.4(4.6)	2005.5(5.6)	66.9(20.5)<br>[8.3(1.9)]<br>173814	90.1(20.4)<br>170809	31.7(6.4)<br>170137
Sulphonylurea	8729284	161652	56.9%	3.5(3.9)<br>144909	63.7(12.0)	6.0(4.9)	2003.9(6.8)	66.7(21.0)<br>[8.2(1.9)]<br>104509	86.6(20.0)<br>102864	30.5(6.3)<br>102283
Thiazolidinedione	1858519	52685	57.7%	6.1(4.6)<br>46644	62.8(11.3)	3.7(3.2)	2006.0(3.2)	69.2(17.6)<br>[8.5(1.6)]<br>42716	90.5(20.4)<br>38334	31.8(6.5)<br>38276
DPP4 inhibitor	1057248	46672	59.0%	8(5.4)<br>42502	64.3(11.5)	2.2(1.9)	2012.0(2.1)	68.3(17.2)<br>[8.4(1.6)]<br>36664	93.5(20.9)<br>33401	32.7(6.6)<br>33335
GLP1-R agonist	284313	12931	56.3%	8.6(4.9)<br>11604	58.9(9.4)	2.3(2.0)	2011.5(2.2)	71.8(18.4)<br>[8.7(1.7)]<br>10490	108.6(22.5)<br>10642	37.8(7.1)<br>10624
SGLT2 inhibitor	77873	8209	60.9%	9.3(5.2)<br>7533	60.3(9.3)	0.8(0.7)	2014.8(0.9)	73.5(16.9)<br>[8.9(1.6)]<br>5952	100.6(21.4)<br>5898	34.7(6.7)<br>5893
Acarbose	197624	7978	52.1%	6.3(4.8)<br>7028	63.8(10.9)	2.7(3.6)	1999.7(4.4)	73.2(20.1)<br>[8.8(1.8)]<br>4284	86.0(20.0)<br>4433	30.7(6.7)<br>4403
Glinide	126288	4572	55.4%	6(4.9)<br>4025	62.6(11.5)	2.8(3.2)	2004.4(4)	69(18.5)<br>[8.5(1.7)]<br>3287	88.1(20.2)<br>3009	31.1(6.5)<br>3002

Insulin	2348351	47122	55.8%	8.6(5.1)<br>41376	65(11.8)	5.4(4.7)	2006.4(5.5)	75.3(19.7)<br>[9.0(1.9)]<br>32284	87.6(20.1)<br>27860	30.9(6.5)<br>27774

Supplementary Table 1: Information on number of prescriptions obtained per drug from application of the inclusion/exclusion criteria. Column 2 N is the number of patients who have at least one prescription for the therapy, descriptives are provided for these patients in the subsequent columns. Mean (SD) or Mean (SD) N (where N is smaller than column 3) reported unless otherwise stated. Baseline taken between 6 months prior to first prescription and 7 days after. *Missing data due to unable to calculate diagnosis date.

CPRD code lists

Diabetes exclusions codes

**medcode**	**readcode**	**desc**
1045	C135.00	Diabetes insipidus
1137	R003.00	[D]Convulsions
1466	C164.00	Polycystic ovaries
2471	K01x100	Nephrotic syndrome in diabetes mellitus
2664	L180900	Gestational diabetes mellitus
8446	L180811	Gestational diabetes mellitus
10098	C10yy00	Other specified diabetes mellitus with other spec
11551	C10B.00	Diabetes mellitus induced by steroids
13279	C104y00	Other specified diabetes mellitus with renal compl
21472	Q441.00	Neonatal diabetes mellitus
22487	C10N.00	Secondary diabetes mellitus
26108	C10B000	Steroid induced diabetes mellitus without complica
30041	C164.13	Multicystic ovaries
30310	K081.00	Nephrogenic diabetes insipidus
30970	Q44B.00	Syndrome of infant of mother with gestational diab
32193	C11y000	Steroid induced diabetes
32999	Q440.00	'Infant of a diabetic mother' syndrome
33343	C10y.00	Diabetes mellitus with other specified manifestati
33969	C10A100	Malnutrition-related diabetes mellitus with ketoac
38617	C101y00	Other specified diabetes mellitus with ketoacidosi
39420	F381300	Myasthenic syndrome due to diabetic amyotrophy
41686	Cyu2000	[X]Other specified diabetes mellitus
46290	C108y00	Other specified diabetes mellitus with multiple co
46624	C10C.11	Maturity onset diabetes in youth
47377	C105y00	Other specified diabetes mellitus with ophthalmic
50064	Q44y100	Transitory metabolic disturbance-infant pre-diabet
51697	C10G.00	Secondary pancreatic diabetes mellitus
52236	C10A.00	Malnutrition-related diabetes mellitus
59288	C103y00	Other specified diabetes mellitus with coma
59991	C10D.11	Maturity onset diabetes in youth type 2
60046	C135.12	Diabetes insipidus - pituitary
61122	C10H.00	Diabetes mellitus induced by non-steroid drugs

61523	C106y00	Other specified diabetes mellitus with neurologica
63371	C10y100	Diabetes mellitus, adult, + other specified manife
64283	C10zy00	Other specified diabetes mellitus with unspecified
66675	C10A000	Malnutrition-related diabetes mellitus with coma
70821	C10yz00	Diabetes mellitus NOS with other specified manifes
93380	C10N100	Cystic fibrosis related diabetes mellitus
94383	C10N000	Secondary diabetes mellitus without complication
94777	ZV13F00	[V]Personal history of gestational diabetes mellit
96506	C10G000	Secondary pancreatic diabetes mellitus without com
98392	C10C.12	Maturity onset diabetes in youth type 1
101172	C135000	Cranial diabetes insipidus
102435	8CE0000	Gestational diabetes information leaflet given
104588	66Ay.00	Gestational diabetes mellitus annual review
106927	PKyP.00	Diab insipidus,diab mell,optic atrophy and deafnes
108013	ZC2CB00	Dietary advice for gestational diabetes
109133	L180700	Pre-existing malnutrition-related diabetes mellitu
109806	8Hgd.00	Discharge from secondary care diabetes service
110481	K081000	Acquired nephrogenic diabetes insipidus
110997	C10y000	Diabetes mellitus, juvenile, + other specified man

Diabetes diagnostic codes

medcode	readcode	desc
506	C100112	Non-insulin dependent diabetes mellitus
608	66A2.00	Follow-up diabetic assessment
711	C10..00	Diabetes mellitus
758	C10F.00	Type 2 diabetes mellitus
1038	C100011	Insulin dependent diabetes mellitus
1323	F420.00	Diabetic retinopathy
1407	C10FJ00	Insulin treated Type 2 diabetes mellitus
1549	C10E.00	Type 1 diabetes mellitus
1647	C108.00	Insulin dependent diabetes mellitus
1682	C101.00	Diabetes mellitus with ketoacidosis
1684	66A4.00	Diabetic on oral treatment
2340	F381311	Diabetic amyotrophy

2342	F372.12	Diabetic neuropathy
2378	66AJ.00	Diabetic - poor control
2379	9N1Q.00	Seen in diabetic clinic
2475	C104.11	Diabetic nephropathy
2478	66AJ100	Brittle diabetes
2986	F420200	Preproliferative diabetic retinopathy
3286	F420100	Proliferative diabetic retinopathy
3550	66A..00	Diabetic monitoring
3837	F420400	Diabetic maculopathy
4513	C109.00	Non-insulin dependent diabetes mellitus
5002	F372.11	Diabetic polyneuropathy
5234	6872.00	Diabetes mellitus screen
5884	C109.11	NIDDM - Non-insulin dependent diabetes mellitus
5905	14O8.00	At risk of diabetes mellitus
6125	66AS.00	Diabetic annual review
6430	9NM0.00	Attending diabetes clinic
6509	C108700	Insulin dependent diabetes mellitus with retinopat
6791	C108800	Insulin dependent diabetes mellitus - poor control
6795	1252.00	FH: Diabetes mellitus
6813	1434.00	H/O: diabetes mellitus
7045	14F4.00	H/O: Admission in last year for diabetes foot prob
7059	8H2J.00	Admit diabetic emergency
7069	F420000	Background diabetic retinopathy
7328	M037200	Cellulitis in diabetic foot
7563	66A3.00	Diabetic on diet only
7777	8H4F.00	Referral to diabetologist
7795	C106.12	Diabetes mellitus with neuropathy
8306	8H7f.00	Referral to diabetes nurse
8403	C109700	Non-insulin dependent diabetes mellitus - poor con
8414	8CA4100	Pt advised re diabetic diet
8618	ZLA2500	Seen by diabetic liaison nurse
8836	66AR.00	Diabetes management plan given
8842	66A5.00	Diabetic on insulin
9013	66AJ.11	Unstable diabetes
9145	9N4I.00	DNA - Did not attend diabetic clinic
9308	ZV18000	[V]Family history of diabetes mellitus
9835	2BBL.00	O/E - diabetic maculopathy present both eyes
9881	M271200	Mixed diabetic ulcer - foot
9897	9OL..00	Diabetes monitoring admin.
9958	42W..00	Hb. A1C - diabetic control
9974	9N1v.00	Seen in diabetic eye clinic
10099	F420300	Advanced diabetic maculopathy
10192	1154200	No significant family history of diabetes

10278	L180800	Diabetes mellitus arising in pregnancy
10418	C10ED00	Type 1 diabetes mellitus with nephropathy
10642	ZC2C800	Dietary advice for diabetes mellitus
10659	F464000	Diabetic cataract
10692	C10EM00	Type 1 diabetes mellitus with ketoacidosis
10755	F420600	Non proliferative diabetic retinopathy
10824	9N1i.00	Seen in diabetic foot clinic
10977	66Ac.00	Diabetic peripheral neuropathy screening
11018	8HBG.00	Diabetic retinopathy 12 month review
11041	9h41.00	Excepted from diabetes qual indicators: Patient un
11094	9NND.00	Under care of diabetic foot screener
11129	2BBQ.00	O/E - left eye background diabetic retinopathy
11149	R102.11	[D]Prediabetes
11348	9h42.00	Excepted from diabetes quality indicators: Informe
11359	L180.00	Diabetes mellitus during pregnancy/childbirth/puer
11433	2BBP.00	O/E - right eye background diabetic retinopathy
11471	8B3l.00	Diabetes medication review
11599	7276.00	Pan retinal photocoagulation for diabetes
11626	F420z00	Diabetic retinopathy NOS
11663	M271100	Neuropathic diabetic ulcer - foot
11677	8H7r.00	Refer to diabetic foot screener
11848	C314.11	Renal diabetes
11930	9NN9.00	Under care of diabetes specialist nurse
11977	ZL62500	Referral to diabetes nurse
12030	9OL6.00	Diabetes monitoring 3rd letter
12213	8BL2.00	Patient on maximal tolerated therapy for diabetes
12225	8H7C.00	Refer, diabetic liaison nurse
12247	8I6G.00	Diabetic foot examination not indicated
12262	8I3X.00	Diabetic retinopathy screening refused
12307	66AU.00	Diabetes care by hospital only
12455	C10E.11	Type I diabetes mellitus
12506	66AP.00	Diabetes: practice programme
12507	9N2i.00	Seen by diabetic liaison nurse
12640	C10FC00	Type 2 diabetes mellitus with nephropathy
12675	66AQ.00	Diabetes: shared care programme
12677	ZV77100	[V]Screening for diabetes mellitus
12682	679R.00	Patient offered diabetes structured education prog
12703	3881.00	Education score - diabetes
12736	C10F500	Type 2 diabetes mellitus with gangrene
13057	679L.00	Health education - diabetes
13067	66AZ.00	Diabetic monitoring NOS
13069	66A8.00	Has seen dietician - diabetes

13070	66A1.00	Initial diabetic assessment
13071	66AI.00	Diabetic - good control
13074	13B1.00	Diabetic diet
13078	13AC.00	Diabetic weight reducing diet
13097	2BBT.00	O/E - right eye proliferative diabetic retinopathy
13099	2BBR.00	O/E - right eye preproliferative diabetic retinopa
13100	2BBJ.00	O/E - no right diabetic retinopathy
13101	2BBV.00	O/E - left eye proliferative diabetic retinopathy
13102	2BBW.00	O/E - right eye diabetic maculopathy
13103	2BBS.00	O/E - left eye preproliferative diabetic retinopat
13104	2BBK.00	O/E - no left diabetic retinopathy
13108	2BBX.00	O/E - left eye diabetic maculopathy
13191	9OL..11	Diabetes clinic administration
13192	9OLA.00	Diabetes monitor. check done
13194	9OL4.00	Diabetes monitoring 1st letter
13195	9OL5.00	Diabetes monitoring 2nd letter
13196	66AD.00	Fundoscopy - diabetic check
13197	9OL1.00	Attends diabetes monitoring
13241	1228.00	No family history diabetes
13245	12G2.00	FH: Diabetes in pregnancy
13678	ZL62600	Referral to diabetic liaison nurse
14049	42WZ.00	Hb. A1C - diabetic control NOS
14050	42c..00	HbA1 - diabetic control
14803	C100100	Diabetes mellitus, adult onset, no mention of comp
14889	C100111	Maturity onset diabetes
15690	C103.00	Diabetes mellitus with ketoacidotic coma
16230	C106.00	Diabetes mellitus with neurological manifestation
16490	66AH.00	Diabetic treatment changed
16491	C106.13	Diabetes mellitus with polyneuropathy
16502	C104.00	Diabetes mellitus with renal manifestation
16881	ZV65312	[V]Dietary counselling in diabetes mellitus
16946	13L4.11	Diabetic child
16971	ZV77111	[V]Screening for diabetes mellitus (DM)
17067	F171100	Autonomic neuropathy due to diabetes
17095	2G5A.00	O/E - Right diabetic foot at risk
17247	F35z000	Diabetic mononeuritis NOS
17262	C109600	Non-insulin-dependent diabetes mellitus with retin
17313	F440700	Diabetic iritis
17545	C108F11	Type I diabetes mellitus with diabetic cataract
17858	C108.12	Type 1 diabetes mellitus
17859	C109.12	Type 2 diabetes mellitus
17869	66AL.00	Diabetic-uncooperative patient

17886	66AM.00	Diabetic - follow-up default
17910	13LZ.11	Diabetic relative
18056	2G5C.00	Foot abnormality - diabetes related
18066	8CE0.00	Diabetic leaflet given
18142	N030000	Diabetic cheiroarthropathy
18143	C109G11	Type II diabetes mellitus with arthropathy
18167	66AT.00	Annual diabetic blood test
18185	2G5D.00	Foot abnormality - non-diabetes
18209	C109012	Type 2 diabetes mellitus with renal complications
18219	C109.13	Type II diabetes mellitus
18230	C108J12	Type 1 diabetes mellitus with neuropathic arthropa
18264	C109J12	Insulin treated Type II diabetes mellitus
18278	C109J00	Insulin treated Type 2 diabetes mellitus
18311	68A7.00	Diabetic retinopathy screening
18387	C10E700	Type 1 diabetes mellitus with retinopathy
18390	C10FM00	Type 2 diabetes mellitus with persistent microalbu
18425	C10FB00	Type 2 diabetes mellitus with polyneuropathy
18496	C10F600	Type 2 diabetes mellitus with retinopathy
18505	C108.11	IDDM-Insulin dependent diabetes mellitus
18642	C10EH00	Type 1 diabetes mellitus with arthropathy
18662	8HBH.00	Diabetic retinopathy 6 month review
18683	C10E500	Type 1 diabetes mellitus with ulcer
18747	8I6F.00	Diabetic retinopathy screening not indicated
18766	212H.00	Diabetes resolved
18777	C10F000	Type 2 diabetes mellitus with renal complications
18824	8I3W.00	Diabetic foot examination declined
19203	1I0..00	Diabetes mellitus excluded
19381	8HTk.00	Referral to diabetic eye clinic
19739	68A9.00	Diabetic retinopathy screening offered
20696	66AA.11	Injection sites - diabetic
20900	9OLA.11	Diabetes monitored
21482	C102.00	Diabetes mellitus with hyperosmolar coma
21689	13AB.00	Diabetic lipid lowering diet
21983	C108012	Type 1 diabetes mellitus with renal complications
22023	66AJz00	Diabetic - poor control NOS
22130	9OL3.00	Diabetes monitoring default
22189	ZV18011	[V]Family history of diabetes mellitus (DM)
22573	C106z00	Diabetes mellitus NOS with neurological manifestat
22823	66Ab.00	Diabetic foot examination
22871	C10EP00	Type 1 diabetes mellitus with exudative maculopath
22884	C10F.11	Type II diabetes mellitus
22967	2BBF.00	Retinal abnormality - diabetes related

23005	1253.00	FH: Diabetes mellitus in first degree relative
23479	C350011	Bronzed diabetes
24327	M271000	Ischaemic ulcer diabetic foot
24363	8A13.00	Diabetic stabilisation
24423	C108.13	Type I diabetes mellitus
24458	C109711	Type II diabetes mellitus - poor control
24490	C100000	Diabetes mellitus, juvenile type, no mention of co
24571	F372200	Asymptomatic diabetic neuropathy
24693	C109G00	Non-insulin dependent diabetes mellitus with arthr
24694	C108B00	Insulin dependent diabetes mellitus with mononeuro
24836	C109C12	Type 2 diabetes mellitus with nephropathy
25041	ZC2CA00	Dietary advice for type II diabetes
25591	C10FQ00	Type 2 diabetes mellitus with exudative maculopath
25627	C10F700	Type 2 diabetes mellitus - poor control
25636	66Aa.00	Diabetic diet - poor compliance
26054	C10FL00	Type 2 diabetes mellitus with persistent proteinur
26603	9OL2.00	Refuses diabetes monitoring
26604	66AY.00	Diabetic diet - good compliance
26605	9OLB.00	Attended diabetes structured education programme
26664	2G5B.00	O/E - Left diabetic foot at risk
26665	2G51100	Foot abnormality - non-diabetes
26666	2G5E.00	O/E - Right diabetic foot at low risk
26667	2G5I.00	O/E - Left diabetic foot at low risk
26855	C108400	Unstable insulin dependent diabetes mellitus
27891	N030100	Diabetic Charcot arthropathy
27921	2G51000	Foot abnormality - diabetes related
28574	9h4..00	Exception reporting: diabetes quality indicators
28622	2126300	Diabetes resolved
28769	66AV.00	Diabetic on insulin and oral treatment
28856	8CP2.00	Transition of diabetes care options discussed
28873	66Ai.00	Diabetic 6 month review
29041	66AN.00	Date diabetic treatment start
29979	C109900	Non-insulin-dependent diabetes mellitus without co
30294	C10EL00	Type 1 diabetes mellitus with persistent microalbu
30323	C10EK00	Type 1 diabetes mellitus with persistent proteinur
30477	F420700	High risk proliferative diabetic retinopathy
30648	9N4p.00	Did not attend diabetic retinopathy clinic
31053	R054300	[D]Widespread diabetic foot gangrene
31141	9OL8.00	Diabetes monitor.phone invite

31156	2G5J.00	O/E - Left diabetic foot at moderate risk
31157	2G5F.00	O/E - Right diabetic foot at moderate risk
31171	2G5G.00	O/E - Right diabetic foot at high risk
31172	2G5K.00	O/E - Left diabetic foot at high risk
31240	9OL7.00	Diabetes monitor.verbal invite
31241	9OLZ.00	Diabetes monitoring admin.NOS
31310	C108900	Insulin dependent diabetes maturity onset
31790	F372.00	Polyneuropathy in diabetes
32359	ZRbH.00	Perceived control of insulin-dependent diabetes
32403	C107.11	Diabetes mellitus with gangrene
32556	C107.12	Diabetes with gangrene
32619	66Af.00	Patient diabetes education review
32627	C10FN00	Type 2 diabetes mellitus with ketoacidosis
32739	9N0n.00	Seen in community diabetes specialist clinic
32770	44V3.00	Glucose tol. test diabetic
33254	C105.00	Diabetes mellitus with ophthalmic manifestation
33807	C107200	Diabetes mellitus, adult with gangrene
34152	G73y000	Diabetic peripheral angiopathy
34268	C10F200	Type 2 diabetes mellitus with neurological complic
34283	C105z00	Diabetes mellitus NOS with ophthalmic manifestatio
34450	C10FK00	Hyperosmolar non-ketotic state in type 2 diabetes
34528	3882.00	Diabetes well being questionnaire
34541	8HVU.00	Private referral to diabetologist
34639	L180100	Diabetes mellitus during pregnancy - baby delivere
34912	C109400	Non-insulin dependent diabetes mellitus with ulcer
35105	C104100	Diabetes mellitus, adult onset, with renal manifes
35107	C104z00	Diabetes mellitus with nephropathy NOS
35116	2G5L.00	O/E - Left diabetic foot - ulcerated
35288	C10E800	Type 1 diabetes mellitus - poor control
35316	2G5H.00	O/E - Right diabetic foot - ulcerated
35321	8H3O.00	Non-urgent diabetic admission
35383	9OLD.00	Diabetic patient unsuitable for digital retinal ph
35385	C10FH00	Type 2 diabetes mellitus with neuropathic arthropa
35399	C107.00	Diabetes mellitus with peripheral circulatory diso
35785	F372100	Chronic painful diabetic neuropathy
36633	C109K00	Hyperosmolar non-ketotic state in type 2 diabetes
36669	66b1.00	Diabetic monitoring not required

36695	C10D.00	Diabetes mellitus autosomal dominant type 2
36855	2BBG.00	Retinal abnormality - non-diabetes
37036	ZV19800	[V]Family history of diabetes mellitus
37315	F3y0.00	Diabetic mononeuropathy
37648	C109J11	Insulin treated non-insulin dependent diabetes mel
37806	C10FF00	Type 2 diabetes mellitus with peripheral angiopath
38078	66A9.00	Understands diet - diabetes
38103	9N0m.00	Seen in diabetic nurse consultant clinic
38129	9N0o.00	Seen in community diabetic specialist nurse clinic
38130	ZRB6.00	Diabetes wellbeing questionnaire
38161	C108711	Type I diabetes mellitus with retinopathy
38986	C100.00	Diabetes mellitus with no mention of complication
39070	C10EE00	Type 1 diabetes mellitus with hypoglycaemic coma
39317	C106100	Diabetes mellitus, adult onset, + neurological man
39809	C108J00	Insulin dependent diab mell with neuropathic arthr
40023	C102000	Diabetes mellitus, juvenile type, with hyperosmola
40401	C109500	Non-insulin dependent diabetes mellitus with gangr
40682	C10E900	Type 1 diabetes mellitus maturity onset
40837	C10EN00	Type 1 diabetes mellitus with ketoacidotic coma
41049	C108712	Type 1 diabetes mellitus with retinopathy
41389	C105100	Diabetes mellitus, adult onset, + ophthalmic manif
41716	C108C00	Insulin dependent diabetes mellitus with polyneuro
42505	C101z00	Diabetes mellitus NOS with ketoacidosis
42567	C103000	Diabetes mellitus, juvenile type, with ketoacidoti
42729	C108E11	Type I diabetes mellitus with hypoglycaemic coma
42762	C109612	Type 2 diabetes mellitus with retinopathy
42831	C10E200	Type 1 diabetes mellitus with neurological complic
43139	C102100	Diabetes mellitus, adult onset, with hyperosmolar
43227	C10F311	Type II diabetes mellitus with multiple complicati
43453	C10C.00	Diabetes mellitus autosomal dominant
43785	C109D00	Non-insulin dependent diabetes mellitus with hypog

43857	C10M.00	Lipoatrophic diabetes mellitus
43921	C10E400	Unstable type 1 diabetes mellitus
43951	66AK.00	Diabetic - cooperative patient
44033	F345000	Diabetic mononeuritis multiplex
44260	C108F00	Insulin dependent diabetes mellitus with diabetic
44312	9M10.00	Informed dissent for diabetes national audit
44440	C108E00	Insulin dependent diabetes mellitus with hypoglyca
44443	C108500	Insulin dependent diabetes mellitus with ulcer
44779	C109E12	Type 2 diabetes mellitus with diabetic cataract
44982	C10FE00	Type 2 diabetes mellitus with diabetic cataract
44993	46Z0.00	Urine screening test for diabetes
45250	ZL22500	Under care of diabetic liaison nurse
45276	C10E312	Insulin dependent diabetes mellitus with multiple
45467	C109B00	Non-insulin dependent diabetes mellitus with polyn
45491	C10z.00	Diabetes mellitus with unspecified complication
45913	C109712	Type 2 diabetes mellitus - poor control
45914	C108812	Type 1 diabetes mellitus - poor control
45919	C109212	Type 2 diabetes mellitus with neurological complic
46150	C109512	Type 2 diabetes mellitus with gangrene
46301	C10EC00	Type 1 diabetes mellitus with polyneuropathy
46521	9N2d.00	Seen by diabetologist
46533	13Y1.00	Diabetic association member
46577	66AX.00	Diabetes: shared care in pregnancy - diabetol and
46850	C108811	Type I diabetes mellitus - poor control
46917	C10FD00	Type 2 diabetes mellitus with hypoglycaemic coma
46963	C108000	Insulin-dependent diabetes mellitus with renal com
47011	8Hj0.00	Referral to diabetes structured education programm
47032	8CS0.00	Diabetes care plan agreed
47058	8Hg4.00	Discharged from care of diabetes specialist nurse
47144	2BBM.00	O/E - diabetic maculopathy absent both eyes
47315	C10F711	Type II diabetes mellitus - poor control
47321	C10F100	Type 2 diabetes mellitus with ophthalmic complicat
47328	2BBk.00	O/E - right eye stable treated prolif diabetic ret
47341	8A12.00	Diabetic crisis monitoring

47370	8HLE.00	Diabetology D.V. done
47409	C109B11	Type II diabetes mellitus with polyneuropathy
47582	C10E000	Type 1 diabetes mellitus with renal complications
47584	F420500	Advanced diabetic retinal disease
47649	C10E100	Type 1 diabetes mellitus with ophthalmic complicat
47650	C10E300	Type 1 diabetes mellitus with multiple complicatio
47816	C109H11	Type II diabetes mellitus with neuropathic arthrop
47954	C10F900	Type 2 diabetes mellitus without complication
48078	F372000	Acute painful diabetic neuropathy
48192	C109E11	Type II diabetes mellitus with diabetic cataract
49074	C10F400	Type 2 diabetes mellitus with ulcer
49146	C108211	Type I diabetes mellitus with neurological complic
49276	C108100	Insulin-dependent diabetes mellitus with ophthalmi
49554	C10EF00	Type 1 diabetes mellitus with diabetic cataract
49559	L180300	Diabetes mellitus during pregnancy - baby not yet
49640	2G5W.00	O/E - left chronic diabetic foot ulcer
49655	C10F611	Type II diabetes mellitus with retinopathy
49869	C109G12	Type 2 diabetes mellitus with arthropathy
49884	6761.00	Diabetic pre-pregnancy counselling
49949	C10E411	Unstable type I diabetes mellitus
50175	66AW.00	Diabetic foot risk assessment
50225	C109011	Type II diabetes mellitus with renal complications
50429	C109100	Non-insulin-dependent diabetes mellitus with ophth
50527	C10FB11	Type II diabetes mellitus with polyneuropathy
50609	L180600	Pre-existing diabetes mellitus, non-insulin-depend
50813	C109A11	Type II diabetes mellitus with mononeuropathy
50937	8HTe.00	Referral to diabetes preconception counselling cli
50960	L180500	Pre-existing diabetes mellitus, insulin-dependent
50972	C100z00	Diabetes mellitus NOS with no mention of complicat
51066	9OLC.00	Family/carer attended diabetes structured educatio
51261	C10E.12	Insulin dependent diabetes mellitus

51756	C10FP00	Type 2 diabetes mellitus with ketoacidotic coma
51957	C108511	Type I diabetes mellitus with ulcer
52041	2BBl.00	O/E - left eye stable treated prolif diabetic reti
52104	C108300	Insulin dependent diabetes mellitus with multiple
52212	Cyu2.00	[X]Diabetes mellitus
52237	9360.00	Patient held diabetic record issued
52283	C108200	Insulin-dependent diabetes mellitus with neurologi
52303	C109000	Non-insulin-dependent diabetes mellitus with renal
52630	2BBo.00	O/E - sight threatening diabetic retinopathy
53200	C101000	Diabetes mellitus, juvenile type, with ketoacidosi
53238	66AG.00	Diabetic drug side effects
53392	C10F911	Type II diabetes mellitus without complication
53634	R054200	[D]Gangrene of toe in diabetic
54008	C10EJ00	Type 1 diabetes mellitus with neuropathic arthropa
54419	918T.00	Diabetes key contact
54600	C10E412	Unstable insulin dependent diabetes mellitus
54601	9NN8.00	Under care of diabetologist
54846	9OL9.00	Diabetes monitoring deleted
54856	C101100	Diabetes mellitus, adult onset, with ketoacidosis
54899	C109F11	Type II diabetes mellitus with peripheral angiopat
55075	C109411	Type II diabetes mellitus with ulcer
55123	66AO.00	Date diabetic treatment stopp.
55239	C10EQ00	Type 1 diabetes mellitus with gastroparesis
55431	L180X00	Pre-existing diabetes mellitus, unspecified
55842	C109200	Non-insulin-dependent diabetes mellitus with neuro
56268	C109D11	Type II diabetes mellitus with hypoglycaemic coma
56448	C108A00	Insulin-dependent diabetes without complication
57278	C10F011	Type II diabetes mellitus with renal complications
57333	N030011	Diabetic cheiropathy
57389	93C4.00	Patient consent given for addition to diabetic reg
57621	C108D00	Insulin dependent diabetes mellitus with nephropat
57723	8HHy.00	Referral to diabetic register
58133	ZLD7500	Discharge by diabetic liaison nurse
58604	C109611	Type II diabetes mellitus with retinopathy
58639	8I57.00	Patient held diabetic record declined

59253	C10FG00	Type 2 diabetes mellitus with arthropathy
59365	C109C00	Non-insulin dependent diabetes mellitus with nephr
59725	C109111	Type II diabetes mellitus with ophthalmic complica
59903	C106.11	Diabetic amyotrophy
60107	C108411	Unstable type I diabetes mellitus
60208	C108J11	Type I diabetes mellitus with neuropathic arthropa
60499	C108600	Insulin dependent diabetes mellitus with gangrene
60699	C109F12	Type 2 diabetes mellitus with peripheral angiopath
60796	C10FL11	Type II diabetes mellitus with persistent proteinu
61021	68AB.00	Diabetic digital retinopathy screening offered
61071	C109D12	Type 2 diabetes mellitus with hypoglycaemic coma
61210	TJ23z00	Adverse reaction to insulins and antidiabetic agen
61344	C108011	Type I diabetes mellitus with renal complications
61461	9M00.00	Informed consent for diabetes national audit
61470	66AI.00	Diabetic monitoring - higher risk albumin excretio
61557	8HKE.00	Diabetology D.V. requested
61670	889A.00	Diab mellit insulin-glucose infus acute myocardial
61829	C108212	Type 1 diabetes mellitus with neurological complic
62107	C109511	Type II diabetes mellitus with gangrene
62146	C109300	Non-insulin-dependent diabetes mellitus with multi
62209	C10EM11	Type I diabetes mellitus with ketoacidosis
62352	C108H11	Type I diabetes mellitus with arthropathy
62384	2G5V.00	O/E - right chronic diabetic foot ulcer
62613	C10EA11	Type I diabetes mellitus without complication
62674	C10FA00	Type 2 diabetes mellitus with mononeuropathy
63017	C108911	Type I diabetes mellitus maturity onset
63357	C107100	Diabetes mellitus, adult, + peripheral circulatory
63412	8CR2.00	Diabetes clinical management plan
63690	C10FR00	Type 2 diabetes mellitus with gastroparesis
63762	C10z100	Diabetes mellitus, adult onset, + unspecified comp
64142	8HI1.00	Referral for diabetic retinopathy screening
64357	C10zz00	Diabetes mellitus NOS with unspecified complicatio

64384	L180z00	Diabetes mellitus in pregnancy/childbirth/puerperi
64446	C108G00	Insulin dependent diab mell with peripheral angiop
64449	C108z00	Unspecified diabetes mellitus with multiple compli
64571	C109C11	Type II diabetes mellitus with nephropathy
64668	C10FJ11	Insulin treated Type II diabetes mellitus
65025	C107z00	Diabetes mellitus NOS with peripheral circulatory
65062	C103z00	Diabetes mellitus NOS with ketoacidotic coma
65267	C10F300	Type 2 diabetes mellitus with multiple complicatio
65463	F420800	High risk non proliferative diabetic retinopathy
65616	C108H00	Insulin dependent diabetes mellitus with arthropat
65684	U602311	[X] Adverse reaction to insulins and antidiabetic
65704	C109412	Type 2 diabetes mellitus with ulcer
66145	C10EN11	Type I diabetes mellitus with ketoacidotic coma
66475	66Ak.00	Diabetic monitoring - lower risk albumin excretion
66872	C108D11	Type I diabetes mellitus with nephropathy
66965	C109H12	Type 2 diabetes mellitus with neuropathic arthropa
67635	L180000	Diabetes mellitus - unspec whether in pregnancy/pu
67664	ZRBa.00	Education score - diabetes
67853	C106000	Diabetes mellitus, juvenile, + neurological manife
67905	C109211	Type II diabetes mellitus with neurological compli
68105	C10EB00	Type 1 diabetes mellitus with mononeuropathy
68390	C108512	Type 1 diabetes mellitus with ulcer
68546	ZRB4.00	Diabetes clinic satisfaction questionnaire
68714	SL23.00	Insulins and antidiabetic poisoning
68792	C10z000	Diabetes mellitus, juvenile type, + unspecified co
68818	ZRB5.11	DTSQ - Diabetes treatment satisfaction questionnai
68843	C103100	Diabetes mellitus, adult onset, with ketoacidotic
68928	TJ23.00	Adverse reaction to insulins and antidiabetic agen
69043	ZC2C900	Dietary advice for type I diabetes
69163	8HTi.00	Referral to multidisciplinary diabetic clinic

69278	C109E00	Non-insulin depend diabetes mellitus with diabetic
69676	C10EA00	Type 1 diabetes mellitus without complication
69748	C105000	Diabetes mellitus, juvenile type, + ophthalmic man
69993	C10E600	Type 1 diabetes mellitus with gangrene
70316	C109112	Type 2 diabetes mellitus with ophthalmic complicat
70448	C107000	Diabetes mellitus, juvenile +peripheral circulator
70766	C108E12	Type 1 diabetes mellitus with hypoglycaemic coma
72320	C109A00	Non-insulin dependent diabetes mellitus with monon
72333	8HME.00	Listed for Diabetology admissn
72345	C102z00	Diabetes mellitus NOS with hyperosmolar coma
72702	C10E812	Insulin dependent diabetes mellitus - poor control
82474	8Hl4.00	Referral to community diabetes specialist nurse
83532	66Ao.00	Diabetes type 2 review
85660	66An.00	Diabetes type 1 review
85991	C10FM11	Type II diabetes mellitus with persistent microalb
91164	ZRB4.11	CSQ - Diabetes clinic satisfaction questionnaire
91646	C10F411	Type II diabetes mellitus with ulcer
91942	C10E311	Type I diabetes mellitus with multiple complicatio
91943	C10EC11	Type I diabetes mellitus with polyneuropathy
93390	9OLH.00	Attended DAFNE diabetes structured education progr
93468	C10EG00	Type 1 diabetes mellitus with peripheral angiopath
93491	9OLJ.00	DAFNE diabetes structured education programme comp
93529	9OLK.00	DESMOND diabetes structured education programme co
93631	9OLL.00	XPERT diabetes structured education programme comp
93657	8Hj4.00	Referral to DESMOND diabetes structured education
93704	8Hj3.00	Referral to DAFNE diabetes structured education pr
93727	C10FE11	Type II diabetes mellitus with diabetic cataract
93854	9OLM.00	Diabetes structured education programme declined

93870	8Hj5.00	Referral to XPERT diabetes structured education pr
93875	C10E712	Insulin dependent diabetes mellitus with retinopat
93878	C10E511	Type I diabetes mellitus with ulcer
93922	C104000	Diabetes mellitus, juvenile type, with renal manif
94011	9OLG.00	Attended XPERT diabetes structured education progr
94186	9OLF.00	Diabetes structured education programme completed
94330	8H4e.00	Referral to diabetes special interest general prac
94647	9Oy..00	Diabetes screening administration
94699	ZRB5.00	Diabetes treatment satisfaction questionnaire
94955	9NiE.00	Did not attend XPERT diabetes structured education
94956	8I84.00	Did not complete XPERT diabetes structured educati
95093	8I83.00	Did not complete DESMOND diabetes structured educa
95094	8I81.00	Did not complete diabetes structured education pro
95124	9Oy0.00	Diabetes screening invitation
95159	9NiD.00	Did not attend DESMOND diabetes structured educati
95343	C10E711	Type I diabetes mellitus with retinopathy
95351	C10FA11	Type II diabetes mellitus with mononeuropathy
95539	C10FS00	Maternally inherited diabetes mellitus
95553	9NiA.00	Did not attend diabetes structured education progr
95636	C10ER00	Latent autoimmune diabetes mellitus in adult
95641	8Hj1.00	Family/carer referral to diabetes structured educa
95813	9N1o.00	Seen in multidisciplinary diabetic clinic
95992	C108A11	Type I diabetes mellitus without complication
95994	66Aq.00	Diabetic foot screen
96142	38DE.00	Cong heart fail, hypertens, age, diab, stroke 2 ri
96235	C10E911	Type I diabetes mellitus maturity onset
96823	L180400	Diabetes mellitus in pueperium - baby previously d
97281	9Nl4.00	Seen by general practitioner special interest in d
97446	C108912	Type 1 diabetes mellitus maturity onset
97474	C108412	Unstable type 1 diabetes mellitus
97809	8I82.00	Did not complete DAFNE diabetes structured educati

97824	ZRB6.11	DWBQ - Diabetes wellbeing questionnaire
97849	C10E912	Insulin dependent diabetes maturity onset
97894	C10EP11	Type I diabetes mellitus with exudative maculopath
98071	C10E112	Insulin-dependent diabetes mellitus with ophthalmi
98616	C10F211	Type II diabetes mellitus with neurological compli
98704	C10E512	Insulin dependent diabetes mellitus with ulcer
98723	C10FD11	Type II diabetes mellitus with hypoglycaemic coma
98954	3883.00	Diabetes treatment satisfaction questionnaire
98978	38DM.00	Age, BP, clinical feat, duration, diabetes 2 strok
99231	C108B11	Type I diabetes mellitus with mononeuropathy
99277	9NiC.00	Did not attend DAFNE diabetes structured education
99311	C10E111	Type I diabetes mellitus with ophthalmic complicat
99628	Kyu0300	[X]Glomerular disorders in diabetes mellitus
99716	C10EE12	Insulin dependent diabetes mellitus with hypoglyca
99719	C10EA12	Insulin-dependent diabetes without complication
99822	38DK.00	Finnish diabetes risk score
100033	U60231E	[X] Adverse reaction to insulins and antidiabetic
100292	Cyu2300	[X]Unspecified diabetes mellitus with renal compli
100347	C10A500	Malnutritn-relat diabetes melitus wth periph circu
100422	8HgC.00	Discharged from diabetes shared care programme
100436	679L000	Education in self management of diabetes
100533	66AQ000	Unsuitable for diabetes year of care programme
100770	C10EF12	Insulin dependent diabetes mellitus with diabetic
100964	C10F111	Type II diabetes mellitus with ophthalmic complica
101177	66At.00	Diabetic dietary review
101190	66AQ100	Declined consent for diabetes year of care program
101311	C10EC12	Insulin dependent diabetes mellitus with polyneuro
101430	1252000	Family history of diabetes mellitus type 1
101455	9OLN.00	Diabetes monitor invitation by SMS (short message

101456	8IAs.00	Diabetic dietary review declined
101728	66As.00	Diabetic on subcutaneous treatment
101735	C10E212	Insulin-dependent diabetes mellitus with neurologi
101801	66At100	Type II diabetic dietary review
101802	1252100	Family history of diabetes mellitus type 2
101834	9h43.00	Excepted from diabetes qual indicators: service un
101881	2BBr.00	Impaired vision due to diabetic retinopathy
102112	C10E611	Type I diabetes mellitus with gangrene
102163	C10ED12	Insulin dependent diabetes mellitus with nephropat
102201	C10FC11	Type II diabetes mellitus with nephropathy
102316	1JL..00	Suspected diabetes mellitus
102434	66Au.00	Diabetic erectile dysfunction review
102490	66Av.00	Diabetic assessment of erectile dysfunction
102611	66At111	Type 2 diabetic dietary review
102620	C10EL11	Type I diabetes mellitus with persistent microalbu
102704	66At000	Type I diabetic dietary review
102740	C108112	Type 1 diabetes mellitus with ophthalmic complicat
102767	67IJ100	Pre-conception advice for diabetes mellitus
102768	9NiZ.00	Did not attend diabetes foot screening
102946	C10E012	Insulin-dependent diabetes mellitus with renal com
103597	1252111	Family history of diabetes mellitus type II
103743	8IE2.00	Diabetes care plan declined
103798	9b92000	Diabetic medicine
103902	C10FG11	Type II diabetes mellitus with arthropathy
103935	1IA..00	No evidence of diabetic nephropathy
104287	8Hlc.00	Referral to community diabetes service
104323	C10F511	Type II diabetes mellitus with gangrene
104374	67D8.00	Provision of diabetes clinical summary
104453	66At011	Type 1 diabetic dietary review
104639	C10FF11	Type II diabetes mellitus with peripheral angiopat
105207	8HTE100	Referral to community diabetes clinic
105302	K08yA00	Proteinuric diabetic nephropathy
105337	C10E811	Type I diabetes mellitus - poor control
105481	14O8000	High risk of diabetes mellitus
105484	66Az.00	High risk of diabetes mellitus annual review
105585	8CMW700	Diabetes clinical pathway
105740	2G5d.00	O/E - Left diabetic foot at increased risk
105741	2G5e.00	O/E - Right diabetic foot at increased risk
105784	C109912	Type 2 diabetes mellitus without complication
105937	8IEQ.00	Referral to community diabetes specialist nurse de

106061	C10FP11	Type II diabetes mellitus with ketoacidotic coma
106218	9m0A.00	Declined diabetic retinopathy screening
106269	9m0..00	Diabetic retinopathy screening administrative stat
106327	9m04.00	Excluded from diabetic retinopathy screening
106328	9m07.00	Excluded diabetc retinop screen as under care opht
106329	9m08.00	Excluded from diabetic retinopathy screening as bl
106332	9m00.00	Eligible for diabetic retinopathy screening
106350	9m05.00	Excluded from diabetic retinopathy screening as mo
106351	9m09.00	Excluded from diabetic retinop screen as no longer
106352	9m06.00	Excluded from diabetic retinopathy screening as de
106360	K27y700	Erectile dysfunction due to diabetes mellitus
106441	9m01.00	Ineligible for diabetic retinopathy screening
106445	9m0E.00	Excluded from diabetic retinopathy screen physical
106528	C10FN11	Type II diabetes mellitus with ketoacidosis
106604	C11y500	Pre-diabetes
106622	38Gj.00	QDiabetes risk calculator
106679	8OA3.00	Provision of written information about diabetes an
106722	9Oy0300	Diabetic foot screening invitation second letter
106723	9Oy0200	Diabetic foot screening invitation first letter
106738	9Oy0000	Diabetic foot screening invitation
106778	9m0C.00	Excluded frm diabetic retinopathy screen as termin
106953	8IEa.00	Referral to DAFNE diabetes structured educn prog d
107361	679L200	Education about diabetes and driving
107414	8I94.00	Diabetes structured education programme not availa
107423	661N400	Diabetes self-management plan review
107452	66o..00	Further diabetic monitoring
107464	66AS000	Diabetes Year of Care annual review
107508	66AH200	Conversion to insulin by diabetes specialist nurse
107554	38Gv.00	Diabetes UK diabetes risk score
107560	67H9.00	Education about lifestyle for risk of diabetes
107597	9m0D.00	Excluded from diabetic retinopthy screen as learn
107603	C10P.00	Diabetes mellitus in remission
107701	C10FK11	Hyperosmolar non-ketotic state in type II diabetes

107739	679L211	Advice about diabetes and driving
107793	9Oy0400	Diabetic foot screening invitation third letter
107824	C10P100	Type II diabetes mellitus in remission
107881	K08yA11	Clinical diabetic nephropathy
108005	C109312	Type 2 diabetes mellitus with multiple complicatio
108007	C108311	Type I diabetes mellitus with multiple complicatio
108360	C10P000	Type I diabetes mellitus in remission
108634	9NJy.00	In-house diabetic foot screening
108724	C10EQ11	Type I diabetes mellitus with gastroparesis
108890	679L300	Diabetic foot care education
108993	661M400	Diabetes self-management plan agreed
109051	C10E612	Insulin dependent diabetes mellitus with gangrene
109103	C109911	Type II diabetes mellitus without complication
109197	C10FH11	Type II diabetes mellitus with neuropathic arthrop
109222	67HA.00	Lifestyle education for diabetes
109520	9m03.00	Eligibility permanently inactive for diabetic reti
109521	9m02.00	Eligibility temporarily inactive for diabetic reti
109628	C10P011	Type 1 diabetes mellitus in remission
109643	66o1.00	Enquiry about diabetic erectile dysfunction declin
109744	8OAK.00	Provsn written information about diabetes & high c
109760	1M8..00	Diabetic peripheral neuropathic pain
109837	C10E011	Type I diabetes mellitus with renal complications
109865	C109B12	Type 2 diabetes mellitus with polyneuropathy
109878	ZC2C911	Diet advice for insulin-dependent diabetes
110056	9m0B.00	Excluded frm diab retinop screen as no currnt cont
110087	8OAH.00	Provision of written information about diabetes an
110344	66o2.00	Diabetic on non-insulin injectable medication
110379	66o5.00	Diabetic on oral treatment and glucagon-like pepti
110393	13B1000	Diabetic carbohydrate counting diet
110400	C108F12	Type 1 diabetes mellitus with diabetic cataract
110409	679l.00	Diabetic injection administration education
110511	67W1.00	Recommendation self-refer for diabetes structured
110611	C10P111	Type 2 diabetes mellitus in remission
111106	C108A12	Type 1 diabetes mellitus without complication

Diabetes medication codes

Product code	Product name	Drug substance	Drug class
479	Acarbose 50mg tablets	Acarbose	Acarbose
5174	Acarbose 100mg tablets	Acarbose	Acarbose
5621	Glucobay 50mg tablets (Bayer Plc)	Acarbose	Acarbose
9105	Glucobay 100mg tablets (Bayer Plc)	Acarbose	Acarbose
35022	Sitagliptin 100mg tablets	Sitagliptin phosphate	DPP4
35462	Januvia 100mg tablets (Merck Sharp & Dohme Ltd)	Sitagliptin phosphate	DPP4
37875	Vildagliptin 50mg tablets	Vildagliptin	DPP4
39149	Galvus 50mg tablets (Novartis Pharmaceuticals UK L	Vildagliptin	DPP4
41204	Saxagliptin 5mg tablets	Saxagliptin hydrochloride	DPP4
41431	Onglyza 5mg tablets (AstraZeneca UK Ltd)	Saxagliptin hydrochloride	DPP4
45775	Saxagliptin 2.5mg tablets	Saxagliptin hydrochloride	DPP4
45821	Onglyza 2.5mg tablets (AstraZeneca UK Ltd)	Saxagliptin hydrochloride	DPP4
46665	Linagliptin 5mg tablets	Linagliptin	DPP4
46716	Trajenta 5mg tablets (Boehringer Ingelheim Ltd)	Linagliptin	DPP4
48401	Sitagliptin 50mg tablets	Sitagliptin phosphate	DPP4
48533	Sitagliptin 25mg tablets	Sitagliptin phosphate	DPP4
50087	Januvia 50mg tablets (Merck Sharp & Dohme Ltd)	Sitagliptin phosphate	DPP4
50124	Januvia 25mg tablets (Merck Sharp & Dohme Ltd)	Sitagliptin phosphate	DPP4
59177	Alogliptin 25mg tablets		DPP4
59809	Alogliptin 6.25mg tablets	Alogliptin benzoate	DPP4
60328	Alogliptin 12.5mg tablets		DPP4
60681	Vipidia 12.5mg tablets (Takeda UK Ltd)		DPP4
60682	Vipidia 25mg tablets (Takeda UK Ltd)		DPP4
62326	Vipidia 6.25mg tablets (Takeda UK Ltd)	Alogliptin benzoate	DPP4
5678	Nateglinide 120mg tablets	Nateglinide	Glinide
5989	Nateglinide 180mg tablets	Nateglinide	Glinide
9707	Repaglinide 1mg tablets	Repaglinide	Glinide
9748	Repaglinide 2mg tablets	Repaglinide	Glinide
9865	Repaglinide 500microgram tablets	Repaglinide	Glinide
11316	NovoNorm 500microgram tablets (Novo Nordisk Ltd)	Repaglinide	Glinide
11321	NovoNorm 1mg tablets (Novo Nordisk Ltd)	Repaglinide	Glinide

11366	NovoNorm 2mg tablets (Novo Nordisk Ltd)	Repaglinide	Glinide
11483	Nateglinide 60mg tablets	Nateglinide	Glinide
15955	Starlix 120mg tablets (Novartis Pharmaceuticals UK	Nateglinide	Glinide
23945	Starlix 60mg tablets (Novartis Pharmaceuticals UK	Nateglinide	Glinide
27125	Starlix 180mg tablets (Novartis Pharmaceuticals UK	Nateglinide	Glinide
35561	Prandin 2mg tablets (Novo Nordisk Ltd)	Repaglinide	Glinide
36774	Prandin 1mg tablets (Novo Nordisk Ltd)	Repaglinide	Glinide
36948	Prandin 0.5mg tablets (Novo Nordisk Ltd)	Repaglinide	Glinide
52203	Enyglid 0.5mg tablets (Consilient Health Ltd)	Repaglinide	Glinide
61925	NovoNorm 500microgram tablets (Waymade Healthcare	Repaglinide	Glinide
35144	Byetta 5micrograms/0.02ml solution for injection 1	Exenatide	GLP1
35149	Exenatide 10micrograms/0.04ml solution for injecti	Exenatide	GLP1
35150	Byetta 10micrograms/0.04ml solution for injection	Exenatide	GLP1
35251	Exenatide 5micrograms/0.02ml solution for injectio	Exenatide	GLP1
40642	Victoza 6mg/ml solution for injection 3ml pre-fill	Liraglutide	GLP1
40693	Liraglutide 6mg/ml solution for injection 3ml pre-	Liraglutide	GLP1
46458	Exenatide 2mg powder and solvent for suspension fo	Exenatide	GLP1
46469	Bydureon 2mg powder and solvent for suspension for	Exenatide	GLP1
55413	Lixisenatide 20micrograms/0.2ml solution for injec		GLP1
55459	Lixisenatide 10micrograms/0.2ml solution for injec		GLP1
55723	Lixisenatide 10micrograms/0.2ml solution for injec		GLP1

55728	Lyxumia 10micrograms/0.2ml solution for injection		GLP1
55729	Lyxumia 20micrograms/0.2ml solution for injection		GLP1
55767	Lyxumia 10micrograms/20micrograms treatment initia		GLP1
62661	Bydureon 2mg powder and solvent for prolonged-rele	Exenatide	GLP1
62904	Exenatide 2mg powder and solvent for suspension fo		GLP1
63336	Trulicity 1.5mg/0.5ml solution for injection pre-f	Dulaglutide	GLP1
63401	Trulicity 0.75mg/0.5ml solution for injection pre-	Dulaglutide	GLP1
63785	Dulaglutide 0.75mg/0.5ml solution for injection pr	Dulaglutide	GLP1
63823	Dulaglutide 1.5mg/0.5ml solution for injection pre	Dulaglutide	GLP1
64622	Bydureon 2mg powder and solvent for suspension for		GLP1
321	INSULIN HUMAN ACTRAPID (NEUTRAL) 40 I/U INJ		INS
322	Humalog 100units/ml solution for injection 1.5ml c	Insulin lispro	INS
1587	Monotard 100units/ml suspension for injection 10ml	Insulin zinc suspension mixed human	INS
1588	Actrapid 100iu/ml Injection (Novo Nordisk Ltd)	Insulin Soluble Human	INS
1592	Actrapid penfill 100 100iu/ml Penfill (Novo Nordis	Insulin Soluble Human	INS
1593	Insulatard penfill 100 100iu/ml Penfill (Novo Nord	Insulin isophane human	INS
1594	Actrapid NovoLet 100units/ml solution for injectio	Insulin soluble human	INS
1595	Insulatard NovoLet 100units/ml suspension for inje	Insulin isophane human	INS
1643	INSULIN NOVO MONOTARD MC 100 I/U INJ		INS
1645	INSULIN NOVO ACTRAPID MC 100 I/U INJ		INS
1649	Human actraphane 100iu/ml Injection (Novo Nordisk	Insulin Soluble Human/Insulin Isophane Human	INS
1805	Mixtard 30/70 100unit/ml Injection (Novo Nordisk L	Insulin Soluble Human/Insulin Isophane Human	INS

1806	Penmix 30/70 100iu/ml Penfill (Novo Nordisk Ltd)	Insulin Soluble Human/Insulin Isophane Human	INS
1839	INSULIN HUMULIN I (ISOPHANE) 100 I/U INJ		INS
1840	Humulin s 100unit/ml Injection (Eli Lilly and Comp	Insulin Soluble Human	INS
1842	Pork velosulin 100unit/ml Injection (Novo Nordisk	Insulin soluble porcine	INS
1843	Pork Insulatard 100units/ml suspension for injecti	Insulin isophane porcine	INS
1844	Ultratard 100units/ml suspension for injection 10m	Insulin zinc suspension crystalline human	INS
1886	Insulatard 100iu/ml GE injection (Novo Nordisk Ltd	Insulin isophane human	INS
2220	Penmix 20/80 Pen (Novo Nordisk Ltd)	Insulin Soluble Human/Insulin Isophane Human	INS
2221	Mixtard 30 NovoLet 100units/ml suspension for inje	Insulin soluble human/Insulin isophane human	INS
2373	INSULIN HUMAN VELOSULIN 100 I/U INJ		INS
2454	Mixtard 30 penfill 100 100iu/ml Penfill (Novo Nord	Insulin Soluble Human/Insulin Isophane Human	INS
2455	Mixtard 20 NovoLet 100units/ml suspension for inje	Insulin soluble human/Insulin isophane human	INS
2456	Mixtard 10 NovoLet 100units/ml suspension for inje	Insulin soluble human/Insulin isophane human	INS
2459	Pork Mixtard 30 100units/ml suspension for injecti	Insulin soluble porcine/Insulin isophane porcine	INS
2808	INSULIN LENTARD INJ		INS
2812	Mixtard 40 NovoLet 100units/ml suspension for inje	Insulin soluble human/Insulin isophane human	INS
2929	Mixtard 30 100iu/ml GE injection (Novo Nordisk Ltd	Insulin Soluble Human/Insulin Isophane Human	INS
3396	Penmix 10/90 Penfill (Novo Nordisk Ltd)	Insulin Soluble Human/Insulin Isophane Human	INS
3439	Penmix 10/90 Pen (Novo Nordisk Ltd)	Insulin Soluble Human/Insulin Isophane Human	INS

3550	Mixtard 40 penfill 100 100iu/ml Penfill (Novo Nord	Insulin Soluble Human/Insulin Isophane Human	INS
3551	Mixtard 20 penfill 100 100iu/ml Penfill (Novo Nord	Insulin Soluble Human/Insulin Isophane Human	INS
4093	Humulin M2 100units/ml suspension for injection 3m	Insulin soluble human/Insulin isophane human	INS
4129	Insulin soluble porcine 100units/ml solution for i	Insulin soluble porcine	INS
4163	Rapitard MC 100unit/ml Injection (Novo Nordisk Ltd	Insulin/Insulin Soluble Porcine	INS
4198	Humulin m3 100unit/ml M3 injection (Eli Lilly and	Insulin Soluble Human/Insulin Isophane Human	INS
4199	Humulin m1 100unit/ml M1 injection (Eli Lilly and	Insulin Soluble Human/Insulin Isophane Human	INS
4247	Insulin isophane porcine 100units/ml suspension fo	Insulin isophane porcine	INS
4248	INSULIN NOVO ULTRATARD MC 100 I/U INJ		INS
4706	Velosulin 100units/ml solution for injection 10ml	Insulin soluble human	INS
4715	Humalog mix 25 25/75 100units/ml Injection (Eli Li	Insulin Lispro/Insulin Lispro Protamine	INS
4760	Humulin i 100unit/ml Injection (Eli Lilly and Comp	Insulin Isophane Human	INS
4784	Lentard mc 100unit/ml Injection (Novo Nordisk Ltd)	Pork Insulin/Insulin Zinc Suspension Mixed Bovine	INS
4790	Mixtard 50 penfill 100 100iu/ml Penfill (Novo Nord	Insulin soluble human/Insulin isophane human	INS
5021	NovoRapid Penfill 100units/ml solution for injecti	Insulin aspart	INS
5214	Insulin lispro 100units/ml solution for injection	Insulin lispro	INS
5250	Insulin biphasic lispro human prb 25:75; 100 units	Insulin Lispro/Insulin Lispro Protamine	INS
5255	Mixtard 10 penfill 100 100iu/ml Penfill (Novo Nord	Insulin Soluble Human/Insulin Isophane Human	INS
5501	Insuman basal 100iu/ml Injection (Aventis Pharma)	Insulin Isophane Human	INS
5845	Mixtard 30 InnoLet 100units/ml suspension for inje	Insulin soluble human/Insulin isophane human	INS

5891	Insulatard FlexPen 100units/ml suspension for inje	Insulin isophane human	INS
5892	NovoRapid FlexPen 100units/ml solution for injecti	Insulin aspart	INS
5933	Mixtard 50 NovoLet 100units/ml suspension for inje	Insulin soluble human/Insulin isophane human	INS
5953	Insulin glargine 100iu/ml Injection	Insulin Glargine	INS
6057	Lantus 100iu/ml Injection (Aventis Pharma)	Insulin Glargine	INS
6061	Novomix 30 30/70 100units/ml Injection (Novo Nordi	Insulin Aspart/Insulin Aspart Protamine	INS
6209	NovoRapid 100units/ml solution for injection 10ml	Insulin aspart	INS
6447	Insulin aspart human pyr 100 iu/ml Injection	Insulin Aspart	INS
6958	Levemir FlexPen 100units/ml solution for injection	Insulin detemir	INS
6965	Levemir Penfill 100units/ml solution for injection	Insulin detemir	INS
7228	NovoMix 30 FlexPen 100units/ml suspension for inje	Insulin aspart/Insulin aspart protamine	INS
7231	Mixtard 30 Penfill 100units/ml suspension for inje	Insulin soluble human/Insulin isophane human	INS
7237	Lantus 100units/ml solution for injection 3ml pre-	Insulin glargine	INS
7266	Lantus 100units/ml solution for injection 3ml cart	Insulin glargine	INS
7267	NovoMix 30 Penfill 100units/ml suspension for inje	Insulin aspart/Insulin aspart protamine	INS
7300	Mixtard 30 100units/ml suspension for injection 10	Insulin soluble human/Insulin isophane human	INS
7318	Humalog 100units/ml solution for injection 3ml car	Insulin lispro	INS
7319	Mixtard 20 Penfill 100units/ml suspension for inje	Insulin soluble human/Insulin isophane human	INS
7349	Actrapid 100units/ml solution for injection 10ml v	Insulin soluble human	INS
7350	Insulin isophane porcine 100units/ml suspension fo	Insulin isophane porcine	INS

7393	Insulin glargine 100units/ml solution for injectio	Insulin glargine	INS
7400	Insulin glargine 100units/ml solution for injectio	Insulin glargine	INS
7402	Lantus 100units/ml solution for injection 10ml via	Insulin glargine	INS
7537	Humulin Zn 100units/ml suspension for injection 10	Insulin zinc suspension crystalline human	INS
7757	INSULIN NEULENTE (ZINC SUSP)(PURIFIED) 100 I/U INJ		INS
7763	INSULIN NEUPHANE (ISOPHANE)(PURIFIED) 100 I/U INJ		INS
7764	INSULIN NEUSULIN (NEUTRAL)(PURIFIED) 100 I/U INJ		INS
7765	INSULIN NEUTRAL (HUMAN) 100 I/U INJ		INS
7771	Human protaphane penfill 100 100unit/ml Penfill (N	Insulin isophane human	INS
7772	Human protaphane 100unit/ml Injection (Novo Nordis	Insulin isophane human	INS
7783	INSULIN ISOPHANE (HUMAN) 100 I/U INJ		INS
7793	HumaJect M3 Pen 100units/ml suspension for injecti	Insulin soluble human/Insulin isophane human	INS
7861	INSULIN HUMULIN S (NEUTRAL) CARTRIDGE 100 I/U		INS
7959	INSULIN MIXTARD 30/70 40 I/U INJ		INS
8118	Humaject i 100iu/ml Pen (Eli Lilly and Company Ltd	Insulin Isophane Human	INS
8203	Penmix 50/50 100iu/ml Penfill (Novo Nordisk Ltd)	Insulin soluble human/Insulin isophane human	INS
8322	Insulin zinc suspension mixed human pyr 100unit/ml	Insulin Zinc Suspension Crystalline Human/Insulin	INS
8354	INSULIN ISOPHANE 70%/NEUTRAL 30% 100 I/U INJ		INS
8376	INSULIN ISOPHANE 100 I/U		INS
8646	INSULIN ZINC CRYSTALLINE susp 100 I/U INJ		INS

8838	INSULIN SEMITARD 40 I/U INJ		INS
8839	INSULIN SEMITARD 100 I/U INJ		INS
8841	Humulin M5 100units/ml suspension for injection 10	Insulin soluble human/Insulin isophane human	INS
8895	Initard 50/50 100unit/ml Injection (Novo Nordisk L	Insulin Isophane Porcine/Insulin Soluble Porcine	INS
9079	INSULIN SOLUBLE 100 I/U INJ		INS
9341	Insulin biphasic isophane human prb 30:70; 100 uni	Insulin Soluble Human/Insulin Isophane Human	INS
9376	Insulin zinc suspension crystalline human pyr 100u	Insulin Zinc Suspension Crystalline Human	INS
9503	Hypurin Bovine Protamine Zinc 100units/ml suspensi	Insulin protamine zinc bovine	INS
9521	Pork Actrapid 100units/ml solution for injection 1	Insulin soluble porcine	INS
9565	HumaJect S Pen 100units/ml solution for injection	Insulin soluble human	INS
9618	Hypurin Porcine 30/70 Mix 100units/ml suspension f	Insulin soluble porcine/Insulin isophane porcine	INS
9737	Insulatard innolet 100iu/ml Injection (Novo Nordis	Insulin isophane human	INS
10001	Humalog Mix50 Pen 100units/ml suspension for injec	Insulin lispro/Insulin lispro protamine	INS
10067	Insulin biphasic aspart human pyr 30:70; 100 units	Insulin Aspart/Insulin Aspart Protamine	INS
10175	Insulin isophane human 100units/ml suspension for	Insulin isophane human	INS
10184	Insulin detemir 100 iu/ml Solution for injection	Insulin Detemir	INS
10207	Insulin isophane human 100units/ml suspension for	Insulin isophane human	INS
10208	Insulatard InnoLet 100units/ml suspension for inje	Insulin isophane human	INS
10225	Lantus 100units/ml solution for injection 3ml Opti	Insulin glargine	INS
10229	Humulin I Pen 100units/ml suspension for injection	Insulin isophane human	INS
10243	Humalog Mix25 100units/ml suspension for injection	Insulin lispro/Insulin lispro protamine	INS

10244	Mixtard 40 Penfill 100units/ml suspension for inje	Insulin soluble human/Insulin isophane human	INS
10245	Mixtard 10 Penfill 100units/ml suspension for inje	Insulin soluble human/Insulin isophane human	INS
10259	Insulin glargine 100units/ml solution for injectio	Insulin glargine	INS
10264	Humalog Pen 100units/ml solution for injection 3ml	Insulin lispro	INS
10277	Humulin M3 100units/ml suspension for injection 3m	Insulin soluble human/Insulin isophane human	INS
10484	Penmix 20/80 Penfill (Novo Nordisk Ltd)	Insulin Soluble Human/Insulin Isophane Human	INS
10545	INSULIN HUMULIN M4 CARTRIDGE 100 I/U		INS
10546	INSULIN HUMULIN M4 100 I/U INJ		INS
10547	Humulin Lente 100units/ml suspension for injection	Insulin zinc suspension mixed human	INS
10566	INSULIN HUMULIN M CARTRIDGE 100 I/U		INS
10572	Insulin soluble bovine 100unit/ml Injection	Insulin Soluble Bovine	INS
10691	INSULIN ISOPHANE (NPH) 100 I/U INJ		INS
10887	Penmix 40/60 100iu/ml Penfill (Novo Nordisk Ltd)	Insulin Soluble Human/Insulin Isophane Human	INS
10910	Humaject m2 100iu/ml M2 pen (Eli Lilly and Company	Insulin Soluble Human/Insulin Isophane Human	INS
10915	Humaject m1 100iu/ml M1 pen (Eli Lilly and Company	Insulin Soluble Human/Insulin Isophane Human	INS
11055	Insulin biphasic isophane human pyr 20:80; 100 uni	Insulin Soluble Human/Insulin Isophane Human	INS
11056	Insulin biphasic isophane human pyr 30:70; 100 uni	Insulin Soluble Human/Insulin Isophane Human	INS
11080	Insulin isophane human prb 100iu/ml Injection	Insulin Isophane Human	INS
11107	Humulin m4 100unit/ml M4 injection (Eli Lilly and	Insulin Soluble Human/Insulin Isophane Human	INS

11337	NovoRapid Novolet 100units/ml solution for injecti	Insulin aspart	INS
12035	Insulin zinc mixed bovine 100units/ml suspension f	Insulin zinc suspension mixed bovine	INS
12060	INSULIN QUICKSOL (SOLUBLE NEUTRAL) 100 I/U INJ		INS
12244	INSULIN ZINC BOVINE susp 100 I/U INJ		INS
12297	Hypurin bovine neutral 100unit/ml Injection (C P P	Insulin Soluble Bovine	INS
12299	Semitard mc 100unit/ml Injection (Novo Nordisk Ltd	Pork Insulin	INS
12638	Insulin soluble human pyr 100unit/ml Injection	Insulin Soluble Human	INS
12654	Insulin soluble human prb 100unit/ml Injection	Insulin Soluble Human	INS
12818	Human Mixtard 50 100units/ml suspension for inject	Insulin soluble human/Insulin isophane human	INS
13277	Mixtard 50 Penfill 100units/ml suspension for inje	Insulin soluble human/Insulin isophane human	INS
13416	Insulin biphasic 100 units/ml Injection	Insulin/Insulin Soluble Porcine	INS
13516	Hypurin bovine isophane 100unit/ml Injection (C P	Insulin isophane bovine	INS
13550	INSULIN BP 100 I/U		INS
13622	Hypurin porcine neutral 100unit/ml Injection (C P	Insulin soluble porcine	INS
13729	Insulin isophane human emp 100unit/ml Injection	Insulin Isophane Human	INS
13819	Hypurin Porcine Isophane 100units/ml suspension fo	Insulin isophane porcine	INS
13837	Insulin biphasic isophane human prb 10:90; 100 uni	Insulin Soluble Human/Insulin Isophane Human	INS
14270	Humalog Mix25 Pen 100units/ml suspension for injec	Insulin lispro/Insulin lispro protamine	INS
14290	Insulatard Penfill 100units/ml suspension for inje	Insulin isophane human	INS
14299	Insulin glulisine 100units/ml solution for injecti	Insulin glulisine	INS
14301	Insulin detemir 100units/ml solution for injection	Insulin detemir	INS
14313	Insulin lispro 100units/ml solution for injection	Insulin lispro	INS

14330	Insulin detemir 100units/ml solution for injection	Insulin detemir	INS
14339	Hypurin Bovine Neutral 100units/ml solution for in	Insulin soluble bovine	INS
14340	Hypurin Bovine Isophane 100units/ml suspension for	Insulin isophane bovine	INS
14345	Apidra 100units/ml solution for injection 3ml cart	Insulin glulisine	INS
14357	Humulin I 100units/ml suspension for injection 3ml	Insulin isophane human	INS
14362	Insulin lispro 100units/ml solution for injection	Insulin lispro	INS
14504	INSULIN HYPURIN PROTAMINE ZINC 100 I/U INJ		INS
14505	Insulin protamine zinc bovine 100units/ml suspensi	Insulin protamine zinc bovine	INS
14506	INSULIN BOVINE PROTAMINE ZINC 100 I/U INJ		INS
14619	Insulin isophane biphasic porcine 30/70 100units/m	Insulin soluble porcine/Insulin isophane porcine	INS
14644	Insulin biphasic isophane human prb 20:80; 100 uni	Insulin Soluble Human/Insulin Isophane Human	INS
14649	Insulin biphasic isophane human pyr 10:90; 100 uni	Insulin Soluble Human/Insulin Isophane Human	INS
14918	Humulin I 100units/ml suspension for injection 10m	Insulin isophane human	INS
14925	Insulin isophane human vial 100unit/ml Sterile sus	Insulin Isophane Human	INS
14928	Insulatard 100units/ml suspension for injection 10	Insulin isophane human	INS
14930	Hypurin Porcine Neutral 100units/ml solution for i	Insulin soluble porcine	INS
14933	Hypurin Porcine Isophane 100units/ml suspension fo	Insulin isophane porcine	INS
14938	Insulin soluble bovine cartridge 100unit/ml Soluti	Insulin Soluble Bovine	INS
14944	Humulin S 100units/ml solution for injection 3ml c	Insulin soluble human	INS
15040	INSULIN MONOPHANE (ISOPHANE) 100 I/U INJ		INS
15199	Insuman comb 25 100iu/ml Injection (Aventis Pharma	Insulin Soluble Human/Insulin Isophane Human	INS

15484	Insulin isophane bovine 100units/ml suspension for	Insulin isophane bovine	INS
15624	INSULIN ISOPHANE (HIGHLY PURIFIED) 100 I/U INJ		INS
15710	Insulin soluble human emp 100unit/ml Injection	Insulin Soluble Human	INS
15961	Insulin isophane human crb 100iu/ml Injection	Insulin Isophane Human	INS
16129	Insulin soluble human 100units/ml solution for inj	Insulin soluble human	INS
16142	Insulin aspart 100units/ml solution for injection	Insulin aspart	INS
16152	Insulin isophane biphasic human 30/70 100units/ml	Insulin soluble human/Insulin isophane human	INS
16160	Humulin M3 Pen 100units/ml suspension for injectio	Insulin soluble human/Insulin isophane human	INS
16209	INSULIN HYPURIN SOLUBLE 100 I/U INJ		INS
16682	Tempulin 100unit/ml Injection (Knoll Ltd)	Insulin zinc suspension mixed bovine	INS
16700	Insulin zinc mixed bovine vial 100unit/ml Sterile	Insulin Zinc Suspension Mixed Bovine	INS
17712	Hypurin Bovine Lente 100units/ml suspension for in	Insulin zinc suspension mixed bovine	INS
17731	Penmix 50/50 100iu/ml Injection (Novo Nordisk Ltd)	Insulin soluble human/Insulin isophane human	INS
17809	Humaject m4 100iu/ml M4 pen (Eli Lilly and Company	Insulin Soluble Human/Insulin Isophane Human	INS
18224	Humalog 100units/ml solution for injection 10ml vi	Insulin lispro	INS
18301	INSULIN SOLUBLE INJ I/U^2		INS
18461	Insulin zinc mixed human 100units/ml suspension fo	Insulin zinc suspension mixed human	INS
18590	Insulin isophane bovine 100units/ml suspension for	Insulin isophane bovine	INS
18592	Insulin soluble bovine 100units/ml solution for in	Insulin soluble bovine	INS
18593	Humalog Mix50 100units/ml suspension for injection	Insulin lispro protamine/Insulin lispro	INS
18645	INSULIN NEUTRAL (PURIFIED) 100 I/U INJ		INS
18931	Insulin zinc crystalline human 100units/ml suspens	Insulin zinc suspension crystalline human	INS

19491	Apidra 100units/ml solution for injection 10ml via	Insulin glulisine	INS
19513	Humulin M3 100units/ml suspension for injection 10	Insulin soluble human/Insulin isophane human	INS
19707	INSULIN HUMULIN S (NEUTRAL SOLUBLE)		INS
19829	INSULIN NOVO MONOTARD MC		INS
19877	Insulin aspart 100units/ml solution for injection	Insulin aspart	INS
19878	Insulin isophane biphasic human 30/70 100units/ml	Insulin soluble human/Insulin isophane human	INS
20195	INSULIN BOVINE PROTAMINE ZINC 40 I/U INJ		INS
20196	INSULIN SOLUBLE 40 I/U INJ		INS
20422	Insuman comb 15 100iu/ml Injection (Aventis Pharma	Insulin isophane human/Insulin soluble human	INS
20671	INSULIN HUM/ACTRAPHANE		INS
20672	INSULIN HUM/ACTRAPID		INS
20995	Hypurin Porcine 30/70 Mix 100units/ml suspension f	Insulin isophane porcine/Insulin soluble porcine	INS
21110	Insulin biphasic isophane human prb 50:50; 100 uni	Insulin Soluble Human/Insulin Isophane Human	INS
21232	Insulin isophane biphasic human 30/70 100units/ml	Insulin soluble human/Insulin isophane human	INS
21235	Humulin S 100units/ml solution for injection 10ml	Insulin soluble human	INS
21347	Penmix 40/60 100iu/ml Injection (Novo Nordisk Ltd)	Insulin Soluble Human/Insulin Isophane Human	INS
21374	Insulin biphasic isophane human prb 40:60; 100 uni	Insulin Soluble Human/Insulin Isophane Human	INS
21395	Insulin biphasic isophane human pyr 40:60; 100 uni	Insulin Soluble Human/Insulin Isophane Human	INS
21422	Insulin isophane biphasic human 40/60 100units/ml	Insulin soluble human/Insulin isophane human	INS

21554	Insuman comb 50 100iu/ml Injection (Aventis Pharma	Insulin soluble human/Insulin isophane human	INS
21583	Apidra 100units/ml solution for injection 3ml pre-	Insulin glulisine	INS
21590	Insulin glulisine 100units/ml solution for injecti	Insulin glulisine	INS
21945	INSULIN PORK INSULATARD		INS
22058	Pur-in mix 15/85 Injection (C P Pharmaceuticals Lt	Insulin Soluble Human/Insulin Isophane Human	INS
22094	INSULIN HUMULIN M2 VIAL		INS
22155	Humaject m5 100iu/ml M5 pen (Eli Lilly and Company	Insulin Soluble Human/Insulin Isophane Human	INS
22161	INSULIN HUMULIN M1 VIAL		INS
22496	INSULIN ZINC LENTE PURIFIED SUSPENSION		INS
22697	Insulin isophane biphasic human 50/50 100units/ml	Insulin soluble human/Insulin isophane human	INS
22806	INSULIN PORK ACTRAPID		INS
22823	INSULIN ISOPHANE (PURIFIED) 100 I/U INJ		INS
22945	Insuman rapid 100iu/ml Injection (Aventis Pharma)	Insulin Soluble Human	INS
22983	Insuman Rapid 100units/ml solution for injection 3	Insulin soluble human	INS
23003	INSULIN ISOPHANE (NPH) 40 I/U		INS
23099	Insulin aspart biphasic 30/70 100units/ml suspensi	Insulin aspart/Insulin aspart protamine	INS
23231	Hypurin Bovine Neutral 100units/ml solution for in	Insulin soluble bovine	INS
23992	Insuman Basal 100units/ml suspension for injection	Insulin isophane human	INS
23993	Insuman Rapid 100units/ml solution for injection 3	Insulin soluble human	INS
24002	Insuman Comb 25 100units/ml suspension for injecti	Insulin soluble human/Insulin isophane human	INS
24485	INSULIN ZINC ANIMAL SUSPENSION		INS
24593	Neutral insulin bovine 100unit/ml Injection	Insulin Soluble Bovine	INS
24722	INSULIN ISOPHANE 50%/NEUTRAL 50% 100 I/U INJ		INS

24795	Insulin aspart biphasic 30/70 100units/ml suspensi	Insulin aspart/Insulin aspart protamine	INS
24800	Hypurin Porcine 30/70 Mix 100units/ml suspension f	Insulin soluble porcine/Insulin isophane porcine	INS
24845	INSULIN PUR-IN ISOPHANE 100 I/U INJ		INS
24846	Pur-in neutral 100unit/ml Injection (C P Pharmaceu	Insulin Soluble Human	INS
24866	INSULIN INSULATARD (LEO RETARD) 40 I/U INJ		INS
24993	Insuman Comb 25 100units/ml suspension for injecti	Insulin soluble human/Insulin isophane human	INS
25006	INSULIN HUMAN ACTRAPID (NEUTRAL)		INS
25133	Insuman Comb 25 100units/ml suspension for injecti	Insulin soluble human/Insulin isophane human	INS
25479	Insulin soluble porcine 100units/ml solution for i	Insulin soluble porcine	INS
25735	Insulin isophane biphasic human 20/80 100units/ml	Insulin soluble human/Insulin isophane human	INS
25736	Insulin isophane biphasic human 10/90 100units/ml	Insulin soluble human/Insulin isophane human	INS
25812	Insulin isophane human 100units/ml suspension for	Insulin isophane human	INS
26060	Insulin lispro 100units/ml solution for injection	Insulin lispro	INS
26098	Hypurin Porcine Neutral 100units/ml solution for i	Insulin soluble porcine	INS
26403	Pur-in mix 25/75 Injection (C P Pharmaceuticals Lt	Insulin Soluble Human/Insulin Isophane Human	INS
26498	Insulin zinc suspension mixed bovine and porcine 1	Pork Insulin/Insulin Zinc Suspension Mixed Bovine	INS
26621	Insulin soluble human crb 100iu/ml Injection	Insulin Soluble Human	INS
26784	INSULIN ZINC SEMILENTE SUSP BP 100 I/U INJ		INS
27177	Insulin biphasic lispro human prb 50:50; 100 units	Insulin Lispro/Insulin Lispro Protamine	INS
27280	Insulin isophane biphasic porcine 30/70 100units/m	Insulin soluble porcine/Insulin isophane porcine	INS
27396	Insulin soluble porcine 100units/ml solution for i	Insulin soluble porcine	INS

27402	Insulin soluble human 100units/ml solution for inj	Insulin soluble human	INS
27461	Insuman Basal 100units/ml suspension for injection	Insulin isophane human	INS
27614	Penmix 30/70 100iu/ml Injection (Novo Nordisk Ltd)	Insulin Soluble Human/Insulin Isophane Human	INS
27911	INSULIN HUMAN ACTRAPID PENFILL		INS
28096	Insulin isophane biphasic human 50/50 100units/ml	Insulin soluble human/Insulin isophane human	INS
28101	Insulin glulisine 100units/ml solution for injecti	Insulin glulisine	INS
28183	Hypurin Porcine Isophane 100units/ml suspension fo	Insulin isophane porcine	INS
28185	Insulin lispro biphasic 25/75 100units/ml suspensi	Insulin lispro/Insulin lispro protamine	INS
28442	Insulin glulisine 100unit/ml Solution for injectio	Insulin Glulisine	INS
28588	Hypurin Bovine Isophane 100units/ml suspension for	Insulin isophane bovine	INS
28723	INSULIN ZINC BOVINE SUSPENSION		INS
28978	INSULIN PUR-IN MIX 15/85 100 I/U INJ		INS
29567	Insulin aspart 100units/ml solution for injection	Insulin aspart	INS
29837	Insulin biphasic isophane human prb 25:75; 100 uni	Insulin Soluble Human/Insulin Isophane Human	INS
29953	Apidra 100units/ml solution for injection 3ml Opti	Insulin glulisine	INS
30209	Actrapid mc 100unit/ml Injection (Arun Products Lt	Insulin soluble porcine	INS
30236	Isophane insulin 100iu/ml Injection	Insulin Isophane Bovine	INS
30686	Insulin isophane porcine 100units/ml suspension fo	Insulin isophane porcine	INS
30819	Insuman Comb 15 100units/ml suspension for injecti	Insulin soluble human/Insulin isophane human	INS
30861	INSULIN ZINC HUMAN SUSPENSION		INS
31205	Insuman Comb 50 100units/ml suspension for injecti	Insulin soluble human/Insulin isophane human	INS
31258	Insulin lispro biphasic 25/75 100units/ml suspensi	Insulin lispro/Insulin lispro protamine	INS

31267	INSULIN PUR-IN MIX 50/50 100 I/U INJ		INS
31465	Exubera 1mg inhalation powder blisters (Pfizer Ltd	Insulin Human	INS
31467	Exubera 3mg inhalation powder blisters (Pfizer Ltd	Insulin human	INS
32053	INSULIN HUMALOG MIX 25		INS
33167	Insulin biphasic isophane human crb 25:75; 100 uni	Insulin Soluble Human/Insulin Isophane Human	INS
33232	Insulin isophane biphasic human 50/50 100units/ml	Insulin soluble human/Insulin isophane human	INS
33966	Insulatard 100unit/ml Injection (Novo Nordisk Ltd)	Insulin Isophane Human	INS
34031	Monotard mc 100unit/ml Injection (Novo Nordisk Ltd	Pork Insulin	INS
34097	Human initard 50/50 100unit/ml Injection (Novo Nor	Insulin Soluble Human/Insulin Isophane Human	INS
35253	Insuman Comb 50 100units/ml suspension for injecti	Insulin soluble human/Insulin isophane human	INS
35260	Levemir InnoLet 100units/ml solution for injection	Insulin detemir	INS
35468	Insuman Basal 100units/ml suspension for injection	Insulin isophane human	INS
35701	Insulin lispro biphasic 50/50 100units/ml suspensi	Insulin lispro/Insulin lispro protamine	INS
36031	Insulin isophane biphasic porcine 30/70 100units/m	Insulin isophane porcine/Insulin soluble porcine	INS
36066	Insulin isophane bovine 100units/ml suspension for	Insulin isophane bovine	INS
36146	Insulin lispro biphasic 50/50 100units/ml suspensi	Insulin lispro protamine/Insulin lispro	INS
36194	Insulin isophane biphasic human 25/75 100units/ml	Insulin soluble human/Insulin isophane human	INS
36355	Insulin human 1mg inhalation powder blisters	Insulin Human	INS
36356	Insulin human 3mg inhalation powder blisters	Insulin human	INS
36430	Insulin soluble human 100units/ml solution for inj	Insulin soluble human	INS
36513	Velosulin cartridge 100unit/ml Injection (Novo Nor	Insulin soluble porcine	INS
36853	Lantus 100units/ml solution for injection 3ml pre-	Insulin glargine	INS

36920	Apidra 100units/ml solution for injection 3ml pre-	Insulin glulisine	INS
38422	Isophane 100iu/ml Injection (Celltech Pharma Europ	Insulin isophane bovine	INS
38986	Humalog KwikPen 100units/ml solution for injection	Insulin lispro	INS
39006	Humalog Mix25 KwikPen 100units/ml suspension for i	Insulin lispro/Insulin lispro protamine	INS
39086	Humalog Mix50 KwikPen 100units/ml suspension for i	Insulin lispro/Insulin lispro protamine	INS
41120	Insulin isophane biphasic human 50/50 100units/ml	Insulin soluble human/Insulin isophane human	INS
41834	Insulin zinc suspension lente 100iu/ml Injection (	Insulin zinc suspension mixed bovine	INS
42395	Humalog Mix25 100units/ml suspension for injection	Insulin lispro/Insulin lispro protamine	INS
42954	Insulin isophane biphasic human 25/75 100units/ml	Insulin soluble human/Insulin isophane human	INS
43950	Humulin I KwikPen 100units/ml suspension for injec	Insulin isophane human	INS
43953	Insulin lispro biphasic 25/75 100units/ml suspensi	Insulin lispro/Insulin lispro protamine	INS
43991	Humulin M3 KwikPen 100units/ml suspension for inje	Insulin soluble human/Insulin isophane human	INS
44251	Insulin zinc suspension mixed porcine 100unit/ml I	Pork Insulin	INS
44378	Insulin isophane biphasic human 25/75 100units/ml	Insulin soluble human/Insulin isophane human	INS
44480	Insuman Comb 25 100units/ml suspension for injecti	Insulin soluble human/Insulin isophane human	INS
45158	Insuman Comb 15 100units/ml suspension for injecti	Insulin soluble human/Insulin isophane human	INS
46001	Insuman Basal 100units/ml suspension for injection	Insulin isophane human	INS
46666	NovoRapid FlexTouch 100units/ml solution for injec	Insulin aspart	INS
47360	Neutral insulin 100unit/ml Injection (Celltech Pha	Insulin Soluble Bovine	INS
47856	Neuphane 100unit/ml Injection (Wellcome Medical Di	Insulin isophane bovine	INS

49108	NovoRapid Penfill 100units/ml solution for injecti	Insulin aspart	INS
49831	Lantus 100units/ml solution for injection 3ml pre-	Insulin glargine	INS
50633	Lantus 100units/ml solution for injection 3ml cart	Insulin glargine	INS
50691	Human Mixtard 20 Penfill 100units/ml suspension fo	Insulin soluble human/Insulin isophane human	INS
51743	NovoRapid Penfill 100units/ml solution for injecti	Insulin aspart	INS
52522	Humalog Mix50 KwikPen 100units/ml suspension for i	Insulin lispro/Insulin lispro protamine	INS
52722	Human Mixtard 30 Penfill 100units/ml suspension fo	Insulin soluble human/Insulin isophane human	INS
52748	Insulatard Penfill 100units/ml suspension for inje	Insulin isophane human	INS
53118	NovoRapid FlexPen 100units/ml solution for injecti	Insulin aspart	INS
53251	NovoRapid Penfill 100units/ml solution for injecti	Insulin aspart	INS
53710	Insulin human 500units/ml solution for injection 2	Insulin human	INS
54462	Insulin biphasic isophane human emp 25:75; 100 uni	Insulin Soluble Human/Insulin Isophane Human	INS
55234	Tresiba FlexTouch 200units/ml solution for injecti	Insulin degludec	INS
55462	Tresiba FlexTouch 100units/ml solution for injecti	Insulin degludec	INS
55517	Insulin isophane human 100units/ml suspension for	Insulin isophane human	INS
55603	Humalog KwikPen 100units/ml solution for injection	Insulin lispro	INS
55618	Levemir FlexPen 100units/ml solution for injection	Insulin detemir	INS
55687	Insulin degludec 100units/ml solution for injectio		INS
55907	Insulin degludec 100units/ml solution for injectio	Insulin degludec	INS
55910	Tresiba Penfill 100units/ml solution for injection	Insulin degludec	INS
56115	Human Actrapid Penfill 100units/ml solution for in	Insulin soluble human	INS
56489	NovoMix 30 Penfill 100units/ml suspension for inje	Insulin aspart/Insulin aspart protamine	INS

56495	Lantus 100units/ml solution for injection 3ml pre-	Insulin glargine	INS
56502	Actrapid Penfill 100units/ml solution for injectio	Insulin soluble human	INS
56691	Insulin degludec 200units/ml solution for injectio		INS
56857	Insulin isophane biphasic human 15/85 100units/ml	Insulin soluble human/Insulin isophane human	INS
57529	Humalog 100units/ml solution for injection 10ml vi	Insulin lispro	INS
57564	Humalog KwikPen 100units/ml solution for injection	Insulin lispro	INS
57620	Humulin M3 100units/ml suspension for injection 10	Insulin soluble human/Insulin isophane human	INS
57622	Humalog Mix50 KwikPen 100units/ml suspension for i	Insulin lispro/Insulin lispro protamine	INS
59500	Insulin isophane human 100units/ml suspension for	Insulin isophane human	INS
59533	NovoRapid FlexPen 100units/ml solution for injecti	Insulin aspart	INS
60933	Humulin M3 100units/ml suspension for injection 10	Insulin soluble human/Insulin isophane human	INS
60938	Mixtard 30 100units/ml suspension for injection 10	Insulin soluble human/Insulin isophane human	INS
60951	Insulin human 100units/ml solution for injection 1		INS
61845	NovoRapid PumpCart 100units/ml solution for inject		INS
62180	Insulin aspart 100units/ml solution for injection		INS
62276	Humulin R 500units/ml solution for injection 20ml	Insulin human	INS
63464	Humalog KwikPen 200units/ml solution for injection	Insulin lispro	INS
63562	Insulin degludec 100units/ml / Liraglutide 3.6mg/m		INS
63679	Hypurin soluble 100iu/ml Injection (C P Pharmaceut	Insulin Soluble Bovine	INS
64354	Toujeo 300units/ml solution for injection 1.5ml pr	Insulin glargine	INS
64460	Insulin glargine 300units/ml solution for injectio		INS

64723	Abasaglar KwikPen 100units/ml solution for injecti	Insulin glargine	INS
64987	Abasaglar 100units/ml solution for injection 3ml c	Insulin glargine	INS
66316	Lantus 100units/ml solution for injection 10ml via	Insulin glargine	INS
66335	Insulin biphasic isophane porcine 50:50; 100 units	Insulin Isophane Porcine/Insulin Soluble Porcine	INS
67230	Lantus 100units/ml solution for injection 3ml pre-	Insulin glargine	INS
67231	NovoRapid FlexPen 100units/ml solution for injecti	Insulin aspart	INS
67266	Mixtard 50 Penfill 100units/ml suspension for inje	Insulin soluble human/Insulin isophane human	INS
67267	Mixtard 30 InnoLet 100units/ml suspension for inje	Insulin soluble human/Insulin isophane human	INS
67279	Pork Insulatard 100units/ml suspension for injecti	Insulin isophane porcine	INS
67313	NovoRapid 100units/ml solution for injection 10ml	Insulin aspart	INS
67324	Humulin M3 100units/ml suspension for injection 3m	Insulin soluble human/Insulin isophane human	INS
23	Metformin 500mg tablets	Metformin hydrochloride	MFN
93	Metformin 850mg tablets	Metformin hydrochloride	MFN
735	Metformin 100mg/ml Oral solution	Metformin Hydrochloride	MFN
2928	METFORMIN HCl 850 MG TAB		MFN
3252	METFORMIN HCl 500 MG TAB		MFN
7048	Metformin 500mg modified-release tablets	Metformin hydrochloride	MFN
7166	Glucophage 500mg tablets (Merck Serono Ltd)	Metformin hydrochloride	MFN
7610	Glucophage 850mg tablets (Merck Serono Ltd)	Metformin hydrochloride	MFN
7815	METFORMIN 800 MG TAB		MFN
11990	Metformin 500mg/5ml oral solution sugar free	Metformin hydrochloride	MFN
16044	Glucophage SR 500mg tablets (Merck Serono Ltd)	Metformin hydrochloride	MFN
16213	METFORMIN 250 MG TAB		MFN
20810	METFORMIN		MFN
25678	Glucamet 500mg Tablet (Opus Pharmaceuticals Ltd)	Metformin hydrochloride	MFN

26258	Glucamet 850mg Tablet (Opus Pharmaceuticals Ltd)	Metformin hydrochloride	MFN
27501	Orabet 500mg Tablet (Lagap)	Metformin hydrochloride	MFN
31146	Metsol 500mg/5ml oral solution (Kappin Ltd)	Metformin hydrochloride	MFN
33087	Metformin 500mg tablets (Actavis UK Ltd)	Metformin hydrochloride	MFN
33674	Metformin 850mg tablets (A A H Pharmaceuticals Ltd	Metformin hydrochloride	MFN
34004	Metformin 500mg tablets (IVAX Pharmaceuticals UK L	Metformin hydrochloride	MFN
34020	Metformin 850mg tablets (IVAX Pharmaceuticals UK L	Metformin hydrochloride	MFN
34135	Metformin 500mg Tablet (M & A Pharmachem Ltd)	Metformin hydrochloride	MFN
34323	Metformin 500mg tablets (A A H Pharmaceuticals Ltd	Metformin hydrochloride	MFN
34504	Metformin 500mg tablets (Wockhardt UK Ltd)	Metformin hydrochloride	MFN
34598	Metformin 500mg tablets (Mylan Ltd)	Metformin hydrochloride	MFN
34697	Metformin 850mg tablets (Wockhardt UK Ltd)	Metformin hydrochloride	MFN
34742	Metformin 850mg tablets (Teva UK Ltd)	Metformin hydrochloride	MFN
34836	Metformin 850mg tablets (Actavis UK Ltd)	Metformin hydrochloride	MFN
34917	Metformin 500mg tablets (Teva UK Ltd)	Metformin hydrochloride	MFN
38355	Metformin 750mg modified-release tablets	Metformin hydrochloride	MFN
38400	Glucophage SR 750mg tablets (Merck Serono Ltd)	Metformin hydrochloride	MFN
39560	Bolamyn SR 500mg tablets (Teva UK Ltd)	Metformin hydrochloride	MFN
39598	Metformin 1g modified-release tablets	Metformin hydrochloride	MFN
39729	Glucophage SR 1000mg tablets (Merck Serono Ltd)	Metformin hydrochloride	MFN
39988	Metformin 500mg oral powder sachets sugar free	Metformin Hydrochloride	MFN
40007	Glucophage 1000mg oral powder sachets (Merck Seron	Metformin hydrochloride	MFN
40110	Glucophage 500mg oral powder sachets (Merck Serono	Metformin hydrochloride	MFN
40233	Metformin 1g oral powder sachets sugar free	Metformin hydrochloride	MFN

42161	Orabet 500mg Tablet (Sandoz Ltd)	Metformin hydrochloride	MFN
43270	Metformin 500mg/5ml oral solution sugar free (Rose	Metformin hydrochloride	MFN
44250	Metformin 500mg/5ml Oral solution (Hillcross Pharm	Metformin hydrochloride	MFN
45581	Metabet SR 500mg tablets (Morningside Healthcare L	Metformin hydrochloride	MFN
46989	Metabet SR 1000mg tablets (Morningside Healthcare	Metformin hydrochloride	MFN
47939	Glucient SR 500mg tablets (Consilient Health Ltd)	Metformin hydrochloride	MFN
48149	Metformin 500mg tablets (Almus Pharmaceuticals Ltd	Metformin hydrochloride	MFN
49502	Glucophage SR 500mg tablets (Mawdsley-Brooks & Com	Metformin hydrochloride	MFN
49738	Metformin 1g modified-release tablets (A A H Pharm	Metformin hydrochloride	MFN
50570	Glucophage SR 500mg tablets (Lexon (UK) Ltd)	Metformin hydrochloride	MFN
50821	Metformin 850mg tablets (Pfizer Ltd)	Metformin hydrochloride	MFN
50970	Metformin 500mg tablets (Bristol Laboratories Ltd)	Metformin hydrochloride	MFN
51080	Metabet SR 1000mg tablets (Actavis UK Ltd)	Metformin hydrochloride	MFN
51135	Metformin 500mg modified-release tablets (A A H Ph	Metformin hydrochloride	MFN
51527	Metformin 500mg tablets (Boston Healthcare Ltd)	Metformin hydrochloride	MFN
52221	Diagemet XL 500mg tablets (Genus Pharmaceuticals L	Metformin hydrochloride	MFN
52442	Metformin 500mg tablets (Pfizer Ltd)	Metformin hydrochloride	MFN
52634	Glucophage SR 500mg tablets (DE Pharmaceuticals)	Metformin hydrochloride	MFN
53478	Metformin 500mg modified-release tablets (Kent Pha	Metformin hydrochloride	MFN
53774	Metabet SR 500mg tablets (Actavis UK Ltd)	Metformin hydrochloride	MFN
53867	Metformin 500mg tablets (Zentiva)	Metformin hydrochloride	MFN
54442	Metformin (roi) 1000mg Tablet	Metformin Hydrochloride	MFN
54898	Metformin 850mg tablets (Almus Pharmaceuticals Ltd	Metformin hydrochloride	MFN
55270	Duformin 500mg Tablet (Dumex Ltd)	Metformin hydrochloride	MFN

55711	Metformin 500mg tablets (Alliance Healthcare (Dist	Metformin hydrochloride	MFN
55739	Metformin 500mg tablets (Tillomed Laboratories Ltd	Metformin hydrochloride	MFN
57147	Bolamyn SR 1000mg tablets (Teva UK Ltd)	Metformin hydrochloride	MFN
57457	Metformin 500mg tablets (Aurobindo Pharma Ltd)	Metformin hydrochloride	MFN
58051	Metformin 500mg/5ml oral solution	Metformin hydrochloride	MFN
58607	Metformin 500mg/5ml oral solution sugar free (Zent	Metformin hydrochloride	MFN
59620	Glucophage SR 500mg tablets (Waymade Healthcare Pl	Metformin hydrochloride	MFN
60074	Metformin 1g modified-release tablets (Waymade Hea	Metformin hydrochloride	MFN
60286	Metformin 500mg/5ml oral suspension	Metformin hydrochloride	MFN
60968	Metformin 500mg modified-release tablets (Actavis	Metformin hydrochloride	MFN
61043	Sukkarto SR 1000mg tablets (Morningside Healthcare	Metformin hydrochloride	MFN
61559	Sukkarto SR 500mg tablets (Morningside Healthcare	Metformin hydrochloride	MFN
62144	Metformin 500mg modified-release tablets (DE Pharm	Metformin hydrochloride	MFN
62265	Metformin 500mg modified-release tablets (Mawdsley	Metformin hydrochloride	MFN
62605	Metformin 850mg tablets (Kent Pharmaceuticals Ltd)	Metformin hydrochloride	MFN
62824	Metformin 1g modified-release tablets (Actavis UK	Metformin hydrochloride	MFN
63045	Metformin 850mg tablets (Relonchem Ltd)	Metformin hydrochloride	MFN
63307	Metformin 1g/5ml oral solution		MFN
64939	Glucient SR 1000mg tablets (Consilient Health Ltd)	Metformin hydrochloride	MFN
65694	Metformin 500mg modified-release tablets (Waymade	Metformin hydrochloride	MFN
65923	Metformin 1g modified-release tablets (Mawdsley-Br	Metformin hydrochloride	MFN
66136	Glucophage SR 1000mg tablets (Waymade Healthcare P	Metformin hydrochloride	MFN
37874	Vildagliptin 50mg / Metformin 850mg tablets	Metformin Hydrochloride/vildagliptin	MFN+DPP4

37902	Vildagliptin 50mg / Metformin 1g tablets	Vildagliptin/Metformin hydrochloride	MFN+DPP4
38551	Eucreas 50mg/1000mg tablets (Novartis Pharmaceutic	Vildagliptin/Metformin hydrochloride	MFN+DPP4
39203	Eucreas 50mg/850mg tablets (Novartis Pharmaceutica	Metformin hydrochloride/Vildagliptin	MFN+DPP4
43619	Metformin 1g / Sitagliptin 50mg tablets	Sitagliptin phosphate/Metformin hydrochloride	MFN+DPP4
43684	Janumet 50mg/1000mg tablets (Merck Sharp & Dohme L	Sitagliptin phosphate/Metformin hydrochloride	MFN+DPP4
50682	Jentadueto 2.5mg/1000mg tablets (Boehringer Ingelh		MFN+DPP4
52445	Linagliptin 2.5mg / Metformin 1g tablets		MFN+DPP4
52449	Linagliptin 2.5mg / Metformin 850mg tablets		MFN+DPP4
54150	Jentadueto 2.5mg/850mg tablets (Boehringer Ingelhe	Metformin hydrochloride/Linagliptin	MFN+DPP4
54891	Saxagliptin 2.5mg / Metformin 1g tablets	Metformin hydrochloride/Saxagliptin hydrochloride	MFN+DPP4
54973	Saxagliptin 2.5mg / Metformin 850mg tablets		MFN+DPP4
56965	Komboglyze 2.5mg/1000mg tablets (AstraZeneca UK Lt	Metformin hydrochloride/Saxagliptin hydrochloride	MFN+DPP4
58865	Komboglyze 2.5mg/850mg tablets (AstraZeneca UK Ltd		MFN+DPP4
59385	Vipdomet 12.5mg/1000mg tablets (Takeda UK Ltd)		MFN+DPP4
60497	Alogliptin 12.5mg / Metformin 1g tablets		MFN+DPP4
60012	Dapagliflozin 5mg / Metformin 1g tablets	Metformin hydrochloride/Dapagliflozin	MFN+SGLT2
60643	Xigduo 5mg/1000mg tablets (AstraZeneca UK Ltd)	Metformin hydrochloride/Dapagliflozin	MFN+SGLT2
63031	Dapagliflozin 5mg / Metformin 850mg tablets		MFN+SGLT2
63929	Canagliflozin 50mg / Metformin 1g tablets		MFN+SGLT2
64743	Canagliflozin 50mg / Metformin 850mg tablets		MFN+SGLT2
65057	Empagliflozin 5mg / Metformin 1g tablets		MFN+SGLT2

65059	Xigduo 5mg/850mg tablets (AstraZeneca UK Ltd)		MFN+SGLT 2
65066	Empagliflozin 12.5mg / Metformin 1g tablets		MFN+SGLT 2
65083	Synjardy 5mg/1000mg tablets (Boehringer Ingelheim	Empagliflozin/Metformin hydrochloride	MFN+SGLT 2
65344	Empagliflozin 5mg / Metformin 850mg tablets	Empagliflozin/Metformin hydrochloride	MFN+SGLT 2
66008	Synjardy 12.5mg/1000mg tablets (Boehringer Ingelhe	Empagliflozin/Metformin hydrochloride	MFN+SGLT 2
66854	Vokanamet 50mg/1000mg tablets (Janssen-Cilag Ltd)		MFN+SGLT 2
66855	Empagliflozin 12.5mg / Metformin 850mg tablets		MFN+SGLT 2
6855	Avandamet 2mg/500mg tablets (GlaxoSmithKline UK Lt	Rosiglitazone maleate/Metformin hydrochloride	MFN+TZD
7325	Avandamet 4mg/1000mg tablets (GlaxoSmithKline UK L	Metformin hydrochloride/Rosiglitazo ne maleate	MFN+TZD
7375	Rosiglitazone 4mg / Metformin 1g tablets	Metformin hydrochloride/Rosiglitazo ne maleate	MFN+TZD
11601	Rosiglitazone 2mg / Metformin 500mg tablets	Rosiglitazone maleate/Metformin hydrochloride	MFN+TZD
11604	Rosiglitazone 1mg / Metformin 500mg tablets	Rosiglitazone maleate/Metformin hydrochloride	MFN+TZD
11609	Metformin with rosiglitazone 500mg + 1mg Tablet	Metformin Hydrochloride/Rosiglitazo ne Maleate	MFN+TZD
11610	Metformin with rosiglitazone 500mg + 2mg Tablet	Metformin Hydrochloride/Rosiglitazo ne Maleate	MFN+TZD
11717	Rosiglitazone 2mg / Metformin 1g tablets	Metformin hydrochloride/Rosiglitazo ne maleate	MFN+TZD
11737	Metformin with rosiglitazone 1000mg + 4mg Tablet	Metformin Hydrochloride/Rosiglitazo ne Maleate	MFN+TZD
11760	Metformin with rosiglitazone 1000mg + 2mg Tablet	Metformin Hydrochloride/Rosiglitazo ne Maleate	MFN+TZD
14164	Avandamet 2mg/1000mg tablets (GlaxoSmithKline UK L	Metformin hydrochloride/Rosiglitazo ne maleate	MFN+TZD

17580	Avandamet 1mg/500mg tablets (GlaxoSmithKline UK Lt	Rosiglitazone maleate/Metformin hydrochloride	MFN+TZD
18220	Pioglitazone 15mg / Metformin 850mg tablets	Pioglitazone hydrochloride/Metformin hydrochloride	MFN+TZD
30316	Metformin with pioglitazone 850mg + 15mg Tablet	Metformin/Pioglitazone	MFN+TZD
31077	Competact 15mg/850mg tablets (Takeda UK Ltd)	Pioglitazone hydrochloride/Metformin hydrochloride	MFN+TZD
54182	Dapagliflozin 10mg tablets	Dapagliflozin	SGLT2
54203	Forxiga 10mg tablets (AstraZeneca UK Ltd)	Dapagliflozin	SGLT2
54265	Dapagliflozin 5mg tablets	Dapagliflozin	SGLT2
54480	Forxiga 5mg tablets (AstraZeneca UK Ltd)	Dapagliflozin	SGLT2
60066	Invokana 100mg tablets (Janssen-Cilag Ltd)		SGLT2
60073	Canagliflozin 100mg tablets		SGLT2
60211	Canagliflozin 100mg tablets	Canagliflozin hemihydrate	SGLT2
60379	Invokana 300mg tablets (Janssen-Cilag Ltd)		SGLT2
60386	Canagliflozin 300mg tablets		SGLT2
60430	Invokana 100mg tablets (Janssen-Cilag Ltd)	Canagliflozin hemihydrate	SGLT2
61756	Empagliflozin 10mg tablets		SGLT2
62172	Empagliflozin 25mg tablets		SGLT2
62760	Jardiance 10mg tablets (Boehringer Ingelheim Ltd)		SGLT2
63516	Forxiga 10mg tablets (Waymade Healthcare Plc)	Dapagliflozin	SGLT2
64217	Jardiance 25mg tablets (Boehringer Ingelheim Ltd)	Empagliflozin	SGLT2
32	Gliclazide 80mg tablets	Gliclazide	SU
547	Glipizide 2.5mg tablets	Glipizide	SU
1253	Chlorpropamide 100mg tablets	Chlorpropamide	SU
1254	Glibenclamide 5mg tablets	Glibenclamide	SU
1847	Chlorpropamide 250mg tablets	Chlorpropamide	SU
1964	Diamicron 80mg tablets (Servier Laboratories Ltd)	Gliclazide	SU
1965	Tolbutamide 500mg tablets	Tolbutamide	SU
2219	Glibenclamide 2.5mg tablets	Glibenclamide	SU
4862	Diabetamide 2.5mg tablets (Ashbourne Pharmaceutica	Glibenclamide	SU
5276	Glimepiride 1mg tablets	Glimepiride	SU
5316	Glimepiride 4mg tablets	Glimepiride	SU

5353	Glimepiride 2mg tablets	Glimepiride	SU
5627	Gliclazide 30mg modified-release tablets	Gliclazide	SU
5636	Glipizide 5mg tablets	Glipizide	SU
6337	Glimepiride 3mg tablets	Glimepiride	SU
7284	Amaryl 2mg tablets (Zentiva)	Glimepiride	SU
7332	Amaryl 1mg tablets (Zentiva)	Glimepiride	SU
7409	Amaryl 3mg tablets (Zentiva)	Glimepiride	SU
7744	Daonil 5mg tablets (Sanofi)	Glibenclamide	SU
7912	Semi-Daonil 2.5mg tablets (Sanofi)	Glibenclamide	SU
8034	Diabinese 100mg Tablet (Pfizer Ltd)	Chlorpropamide	SU
8168	Diabinese 250mg Tablet (Pfizer Ltd)	Chlorpropamide	SU
8976	Euglucon 2.5mg tablets (Aventis Pharma)	Glibenclamide	SU
9108	TOLBUTAMIDE 250 MG TAB		SU
10427	Tolazamide 250mg Tablet	Tolazamide	SU
11284	Amaryl 4mg tablets (Zentiva)	Glimepiride	SU
11695	Diamicron 30mg MR tablets (Servier Laboratories Lt	Gliclazide	SU
11946	Tolbutamide 50mg/ml Injection	Tolbutamide	SU
12245	Glutril 25mg Tablet (Roche Products Ltd)	Glibornuride	SU
12259	Glibornuride 25mg Tablet	Glibornuride	SU
12455	Rastinon 500mg Tablet (Hoechst Marion Roussel)	Tolbutamide	SU
12513	Glibenese 5mg tablets (Pfizer Ltd)	Glipizide	SU
13331	Euglucon 5mg tablets (Sanofi)	Glibenclamide	SU
15374	Gliclazide 40mg/5ml oral suspension	Gliclazide	SU
16211	TOLBUTAMIDE 100 MG TAB		SU
16602	Calabren 2.5mg Tablet (Berk Pharmaceuticals Ltd)	Glibenclamide	SU
17343	Gliclazide 80mg tablets (A A H Pharmaceuticals Ltd	Gliclazide	SU
17698	Minodiab 5mg tablets (Pfizer Ltd)	Glipizide	SU
17706	Minodiab 2.5mg tablets (Pfizer Ltd)	Glipizide	SU
19336	Tolazamide 100mg Tablet	Tolazamide	SU
21424	Glibenclamide 5mg/5ml oral suspension	Glibenclamide	SU
21489	Tolanase 250mg Tablet (Pharmacia Ltd)	Tolazamide	SU

21564	Gliclazide 80mg tablets (Wockhardt UK Ltd)	Gliclazide	SU
21832	Diabetamide 5mg tablets (Ashbourne Pharmaceuticals	Glibenclamide	SU
21892	Diaglyk 80mg tablets (Ashbourne Pharmaceuticals Lt	Gliclazide	SU
22145	Tolanase 100mg Tablet (Pharmacia Ltd)	Tolazamide	SU
22614	DAONIL 10 MG TAB		SU
22636	TOLBUTAMIDE 1 GM TAB		SU
22858	Acetohexamide 500mg tablets	Acetohexamide	SU
24848	Glymidine sodium 500mg Tablet	Glymidine Sodium	SU
25636	Libanil 2.5mg Tablet (Approved Prescription Servic	Glibenclamide	SU
26118	Dimelor 500mg Tablet (Eli Lilly and Company Ltd)	Acetohexamide	SU
26218	Calabren 5mg Tablet (Berk Pharmaceuticals Ltd)	Glibenclamide	SU
27969	Glymese 250mg Tablet (DDSA Pharmaceuticals Ltd)	Chlorpropamide	SU
28708	Malix 2.5mg Tablet (Lagap)	Glibenclamide	SU
29326	Glipizide 5mg tablets (Mylan Ltd)	Glipizide	SU
29939	Gliclazide 80mg tablets (Mylan Ltd)	Gliclazide	SU
30460	Malix 5mg Tablet (Lagap)	Glibenclamide	SU
31212	Gliclazide 80mg tablets (Actavis UK Ltd)	Gliclazide	SU
31474	Libanil 5mg Tablet (Approved Prescription Services	Glibenclamide	SU
33562	Duclazide 80mg Tablet (Dumex Ltd)	Gliclazide	SU
33673	Tolbutamide 500mg tablets (Actavis UK Ltd)	Tolbutamide	SU
34399	Gliclazide 80mg tablets (IVAX Pharmaceuticals UK L	Gliclazide	SU
34507	Glibenclamide 2.5mg tablets (Wockhardt UK Ltd)	Glibenclamide	SU
34563	Glibenclamide 5mg tablets (Wockhardt UK Ltd)	Glibenclamide	SU
34676	Glibenclamide 2.5mg tablets (A A H Pharmaceuticals	Glibenclamide	SU
34706	Glibenclamide 2.5mg tablets (IVAX Pharmaceuticals	Glibenclamide	SU
34802	Glipizide 5mg tablets (IVAX Pharmaceuticals UK Ltd	Glipizide	SU

34932	Gliclazide 80mg tablets (Genus Pharmaceuticals Ltd	Gliclazide	SU
34957	Tolbutamide 500mg tablets (A A H Pharmaceuticals L	Tolbutamide	SU
36856	Gliclazide 80mg tablets (Sandoz Ltd)	Gliclazide	SU
40365	Glimepiride 1mg tablets (Actavis UK Ltd)	Glimepiride	SU
40425	Nazdol MR 30mg tablets (Teva UK Ltd)	Gliclazide	SU
41558	Glibenclamide 5mg tablets (Teva UK Ltd)	Glibenclamide	SU
41559	Glibenclamide 5mg tablets (A A H Pharmaceuticals L	Glibenclamide	SU
41593	Glibenclamide 2.5mg tablets (Teva UK Ltd)	Glibenclamide	SU
41898	GLIBENCLAMIDE		SU
42790	Gliclazide 80mg Tablet (Merck Generics (UK) Ltd)	Gliclazide	SU
43065	Gliclazide 40mg tablets	Gliclazide	SU
43465	Zicron 40mg tablets (Bristol Laboratories Ltd)	Gliclazide	SU
44304	Glyconon 500mg Tablet (DDSA Pharmaceuticals Ltd)	Tolbutamide	SU
44473	Edicil MR 30mg tablets (Teva UK Ltd)	Gliclazide	SU
44738	Niddaryl 1mg tablets (Dee Pharmaceuticals Ltd)	Glimepiride	SU
45215	Gliclazide 80mg Tablet (Neo Laboratories Ltd)	Gliclazide	SU
45831	Dacadis MR 30mg tablets (Mylan Ltd)	Gliclazide	SU
46927	Tolbutamide 500mg tablets (Teva UK Ltd)	Tolbutamide	SU
47074	Gliclazide 80mg/5ml oral suspension	Gliclazide	SU
47894	Nazdol MR 30mg tablets (Consilient Health Ltd)	Gliclazide	SU
48056	Gliclazide 80mg tablets (Sovereign Medical Ltd)	Gliclazide	SU
51955	Gliclazide 80mg tablets (Accord Healthcare Ltd)	Gliclazide	SU
53288	Gliclazide 30mg modified-release tablets (A A H Ph	Gliclazide	SU
54764	Gliclazide 80mg tablets (Arrow Generics Ltd)	Gliclazide	SU
55862	Gliclazide Oral solution	Gliclazide	SU
56008	Gliclazide 80mg tablets (Almus Pharmaceuticals Ltd	Gliclazide	SU

56437	Gliclazide 60mg modified-release tablets	Gliclazide	SU
57601	Daonil 5mg tablets (Dowelhurst Ltd)	Glibenclamide	SU
57830	Gliclazide 30mg modified-release tablets (Alliance	Gliclazide	SU
58882	Gliclazide 120mg/5ml oral suspension		SU
60495	Gliclazide 80mg tablets (Teva UK Ltd)	Gliclazide	SU
61311	Glimepiride 4mg tablets (Sigma Pharmaceuticals Plc	Glimepiride	SU
61957	Gliclazide 40mg tablets (A A H Pharmaceuticals Ltd	Gliclazide	SU
62014	Glimepiride 2mg tablets (Accord Healthcare Ltd)	Glimepiride	SU
62034	Laaglyda MR 60mg tablets (Consilient Health Ltd)	Gliclazide	SU
63048	Gliclazide 80mg tablets (Alliance Healthcare (Dist	Gliclazide	SU
63131	Ziclaseg 30mg modified-release tablets (Lupin (Eur	Gliclazide	SU
66399	Glimepiride 2mg tablets (A A H Pharmaceuticals Ltd	Glimepiride	SU
67056	Amaryl 1mg tablets (Lexon (UK) Ltd)	Glimepiride	SU
56376	Rosiglitazone 4mg with glimepiride 4mg tablet	Rosiglitazone Maleate/Glimepiride	SU+TZD
469	Rosiglitazone 4mg tablets	Rosiglitazone maleate	TZD
548	Pioglitazone 15mg tablets	Pioglitazone hydrochloride	TZD
5227	Rosiglitazone 8mg tablets	Rosiglitazone maleate	TZD
9662	Avandia 4mg tablets (GlaxoSmithKline UK Ltd)	Rosiglitazone maleate	TZD
9699	Pioglitazone 30mg tablets	Pioglitazone hydrochloride	TZD
10051	Pioglitazone 45mg tablets	Pioglitazone hydrochloride	TZD
13628	Romozin 400mg Tablet (Glaxo Wellcome UK Ltd)	Troglitazone	TZD
15232	Avandia 8mg tablets (GlaxoSmithKline UK Ltd)	Rosiglitazone maleate	TZD
19472	Actos 45mg tablets (Takeda UK Ltd)	Pioglitazone hydrochloride	TZD
20287	Actos 15mg tablets (Takeda UK Ltd)	Pioglitazone hydrochloride	TZD
20889	Actos 30mg tablets (Takeda UK Ltd)	Pioglitazone hydrochloride	TZD
37617	Rosiglitazone 2mg tablet	Rosiglitazone Maleate	TZD

48120	Avandia 2mg Tablet (GlaxoSmithKline UK Ltd)	Rosiglitazone Maleate	TZD
48139	Pioglitazone 30mg tablets (A A H Pharmaceuticals L	Pioglitazone hydrochloride	TZD
56208	Pioglitazone 15mg tablets (A A H Pharmaceuticals L	Pioglitazone hydrochloride	TZD
56831	Troglitazone 200mg Tablet		TZD
57659	Pioglitazone 30mg tablets (Actavis UK Ltd)	Pioglitazone hydrochloride	TZD
62426	Pioglitazone 30mg tablets (Accord Healthcare Ltd)	Pioglitazone hydrochloride	TZD
63046	Pioglitazone 45mg tablets (A A H Pharmaceuticals L	Pioglitazone hydrochloride	TZD
63107	Pioglitazone 45mg tablets (Waymade Healthcare Plc)	Pioglitazone hydrochloride	TZD
63421	Pioglitazone 30mg tablets (Teva UK Ltd)	Pioglitazone hydrochloride	TZD
64900	Glidipion 30mg tablets (Actavis UK Ltd)	Pioglitazone hydrochloride	TZD
65562	Pioglitazone 30mg tablets (Alliance Healthcare (Di	Pioglitazone hydrochloride	TZD
65563	Pioglitazone 15mg tablets (Alliance Healthcare (Di	Pioglitazone hydrochloride	TZD

HbA1c

Records matching the following HbA1c-related medcodes were extracted:

medcode	readcode
5717	42W..11
9958	42W..00
13597	42W1.00
13604	42W3.00
14049	42WZ.00
14050	42c..00
14051	44TB.00
14052	42W..12
14053	42W4.00
19807	42c3.00
27040	44TC.00
29218	42W2.00

37035	66Ae.00
39205	44TL.00
40463	42c1.00
42360	42c0.00
46079	42c2.00
96968	42W5.00
101208	66Ae000
